# Flexible Reference Frames for Grasp Planning in Human Parietofrontal Cortex^1^,^2^,^3^

**DOI:** 10.1523/ENEURO.0008-15.2015

**Published:** 2015-06-24

**Authors:** Frank T. M. Leoné, Simona Monaco, Denise Y. P. Henriques, Ivan Toni, W. Pieter Medendorp

**Affiliations:** 1 Donders Institute for Brain, Cognition and Behavior, Radboud University Nijmegen, 6525 HR, Nijmegen, The Netherlands; 2 Centre for Vision Research, York University, Toronto, Ontario, M3J 1P3 Canada

**Keywords:** functional magnetic resonance imaging, grasping, multivariate, parietofrontal cortex, reference frames, transformations

## Abstract

Reaching to a location in space is supported by a cortical network that operates in a variety of reference frames. Computational models and recent fMRI evidence suggest that this diversity originates from neuronal populations dynamically shifting between reference frames as a function of task demands and sensory modality. In this human fMRI study, we extend this framework to nonmanipulative grasping movements, an action that depends on multiple properties of a target, not only its spatial location. By presenting targets visually or somaesthetically, and by manipulating gaze direction, we investigate how information about a target is encoded in gaze- and body-centered reference frames in dorsomedial and dorsolateral grasping-related circuits. Data were analyzed using a novel multivariate approach that combines classification and cross-classification measures to explicitly aggregate evidence in favor of and against the presence of gaze- and body-centered reference frames. We used this approach to determine whether reference frames are differentially recruited depending on the availability of sensory information, and where in the cortical networks there is common coding across modalities. Only in the left anterior intraparietal sulcus (aIPS) was coding of the grasping target modality dependent: predominantly gaze-centered for visual targets and body-centered for somaesthetic targets. Left superior parieto-occipital cortex consistently coded targets for grasping in a gaze-centered reference frame. Left anterior precuneus and premotor areas operated in a modality-independent, body-centered frame. These findings reveal how dorsolateral grasping area aIPS could play a role in the transition between modality-independent gaze-centered spatial maps and body-centered motor areas.

## Significance Statement

Computational models of sensorimotor control suggest that neuronal populations dynamically shift between reference frames dependent on task demands and sensory modality. This fMRI study distinguished the reference frames used to plan a reach-to-grasp visual or somaesthetic target. Using a novel analysis approach, combining multivariate classification and cross-classification evidence, we distinguished between gaze- and body-centered frames in dorsomedial and dorsolateral grasp circuits. While most parietal regions in these circuits use modality-independent, gaze-centered coordinates and premotor regions use modality-independent, body-centered coordinates, the anterior intraparietal area (aIPS) switches its reference frame dynamically, depending on the sensory modality of the grasp target. We conclude that aIPS operates as an important hub in the transition between modality-independent, gaze-centered spatial maps and body-centered grasp areas.

## Introduction

Parietofrontal neurons involved in reaching to a location in space operate in a variety of reference frames (Battaglia-Mayer et al., 2003; McGuire and Sabes, 2011; Buchholz et al., 2013). Theoretical modeling and behavioral evidence has suggested that these reference frames can be dynamically weighted according to task demands and sensory input (Pouget and Snyder, 2000; Beurze et al., 2007; McGuire and Sabes, 2009).

Recent neurophysiological work (Bernier and Grafton, 2010) has shown how sensory input influences the contributions of different parietofrontal regions while subjects reached out, by rotating their wrist, to touch visual or proprioceptive targets with their right index finger. By changing the fixation point relative to the target, that study showed that the anterior precuneus encoded the motor goal for visual targets selectively in gaze-centered (GC) coordinates, while other parietofrontal areas showed a mixture of GC and body-centered (BC) encoding. In contrast, reaching to proprioceptive targets revealed negligible gaze-centered encoding but considerable body-centered encoding throughout the parietofrontal network. Building on this work, here we investigate how task demands affect these modality-dependent reference frames within the grasping network.

In the study by Bernier and Grafton (2010), “touching” served as the steering task demand. Another common task demand supported by a reaching movement is grasping an object. So far, reaching to grasp has been studied predominantly in the context of visually guided movements. This has led to the notion that reaching to grasp is guided through two visuomotor channels in the parietofrontal network, which are involved in specifying where to move the hand in space and how to shape the fingers around the grasping target (Jeannerod, 1988; Culham and Valyear, 2006; Filimon, 2010). These parietofrontal areas are anatomically organized in a dorsomedial and a dorsolateral circuit (Rizzolatti and Matelli, 2003; Grol et al., 2007; Gamberini et al., 2009), with different accounts emphasizing either the parallel or the hierarchical organizations of those circuits (Culham and Valyear, 2006; Glover, 2004; Cavina-Pratesi et al., 2010b; Grafton, 2010; Verhagen et al., 2013; Binkofski and Buxbaum, 2013). However, these accounts have largely ignored how grasp demands affect the frames of reference involved in specifying where to reach, which could be crucial in understanding the functional contributions of these circuits to the overall sensorimotor transformation (Crawford et al., 2011).

Using fMRI in human participants, we tested the dominant reference frames in the dorsomedial and dorsolateral pathways when preparing a reach to grasp. Toward this end, we manipulated the availability of visual or proprioceptive information about the grasping target (the task demand), and the position of a target in relation to the subject’s gaze and body midline. The manipulation of the sensory modality relies on the rationale that the acquisition of visual information is intimately linked to the direction of gaze, whereas proprioceptive information is linked to the relative body part in the early stages of processing.

Accordingly, this manipulation quantifies how cortical reaching areas adjust to the different frames of reference imposed by sensory information of the grasp demand. The gaze manipulation directly discriminates between activity for reaching to grasp independent from gaze (i.e., linked to the body) and activity linked to gaze direction. We combine these manipulations within a novel multivariate analysis framework, aggregating evidence from both classification and cross-classification measures in favor and against gaze- and body-centered reference frames. Using this novel approach, we unveil reach-to-grasp areas that determine reference frames flexibly, depending on sensory modality, and those that use a modality-independent code.

## Materials and Methods

Nineteen healthy right-handed participants with normal or corrected-to-normal vision participated in this study. The data of one participant (male) were excluded from further analysis due to noncompliance with task instructions. The remaining 18 participants (5 female) were in the age range 18-42 years (mean age, 26.5 years). Participants gave their written consent in accordance with the local ethics committee.

### Experimental setup

**Figure 1:**
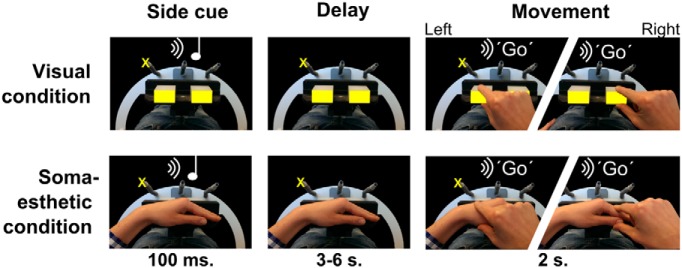
Stimulus setup and experimental paradigm. A rotating platform presented either two visual objects (yellow rectangles, top row) or the participant’s left hand (bottom row, the hand was not visible during the experiment). At the start of the trial (left column), participants were instructed by an auditory tone to plan the grasping movement to either one of the two visual objects, or to one of two segments of the left hand, while fixating one of three fixation lights (the yellow cross marks one of the three possible locations). After a variable delay (middle column), an auditory instruction (right column) triggered the execution of the movement (to the one or the other visual object or hand segment, indicated by jagged images). The experiment was performed in the dark; only elements indicated in yellow were visible to the participant.

Participants were lying supine in the scanner in complete darkness. Their upper body was cushioned and strapped to minimize trunk movement. The head was stabilized with foam blocks and wedges inside a phased-array receiver head coil. Head and coil were tilted 30° above the horizontal plane to allow direct vision of the grasping device. The grasping device was supported by an arch placed above the hips, and consisted of three light-emitting diodes (LEDs; called fixation LEDs) and a rotating platform, which was aligned parallel to the longitudinal body axis (Fig. 1). The fixation LEDs were positioned to the left, at the center, and to the right of the platform, 10 cm above the center of the platform (at −11°, 0°, and 11° of visual angle from the mid-sagittal plane). One half (Fig. 1, top) of the platform supported two Plexiglass blocks [size, 10 × 10 × 5 cm (length × width × height)], 4.5 cm left and right from the central fixation LED. The two Plexiglass blocks were independently illuminated by internal LEDs. The other half of the platform served as a support for the left hand of the subject (Fig. 1, bottom). Between runs, the platform was rotated by the subject to bring either half in view, below the fixation LEDs.

 In particular, the visual blocks were brought in front in the visual condition and were illuminated, and the left hand was positioned next to the subject on the scanner bed. By keeping the left hand away from the visual blocks and the body midline, we guaranteed there was no effect of left hand position on the visual spatial coding.

During the somaesthetic condition, the two visual blocks were turned off and the empty half of the platform was brought to the front to allow the participant to position the left hand on it, with the metacarpo-phalangeal joints aligned on the central fixation LED. This configuration ensured that the wrist and the proximal interphalangeal joint of the index finger were also ∼4.5 cm left and right from the central fixation LED, as the two blocks in the visual condition. The heights of the two grasping locations [left, the base of the thumb (thenar eminence); right, the top of the thumb and the first joint of the index finger] were comparable to the height of the blocks.

In both visual and somaesthetic conditions, the subjects were cued through an auditory instruction to grasp either the left or right grasping target. Participants wore ear phones for the presentation of auditory instructions. Visual stimuli and auditory cues were controlled using Presentation software (version 14.7; Neurobehavioral Systems).

Grasping movements were made with the right hand. Before task initiation, the task was practiced outside the scanner until both grasping and eye movements were performed in accordance with the task. To record reaction and movement time, each movement started from and ended with the right hand on a button box, which was placed on the chest. An fMRI-compatible infrared camera (MRC Systems) recorded the movement of the right hand, allowing for the screening of incorrect target selection or other errors. Trials were only regarded as correct when the correct target was grasped, in between the fixation lights, using a noninterrupted smooth grasping movement. We could not use an MR compatible eye tracker because the present grasp apparatus blocked its field of view. However, eye tracking outside the scanner showed that the subjects could successfully follow the task instructions, fixating when necessary and moving the eyes when instructed (Selen and Medendorp, 2011).

### Experimental paradigm

Participants performed an instructed-delay grasping task to either visual or somaesthetic targets, which were presented to the left or right of the body midline (see description above), while fixating on one of the three fixation LEDs (Fig. 1*B*). Participants started each trial with their right hand on the button box and their gaze directed at one of the three fixation LEDs. An auditory cue (160 or 480 Hz) indicated the target of the ensuing movement (sound–target mapping was counterbalanced across subjects). After a variable delay period (range, 3-6 s; uniform distribution), an auditory spoken “Go” cue instructed the participant to execute the movement, grasping the target and then returning to the button box. The grasp was nonmanipulative (i.e., firmly touching the target with all fingers), using the grasp equivalent of the manipulation used in Bernier and Grafton (2010). Subsequently, the fixation point changed position twice, as follows: in first 2 s after the Go cue, or 500 ms after movement onset, whichever was later; and then again 1 s later. Finally, 500 ms after the second saccade, the next trial started with the presentation of a new auditory cue. The double change in gaze position was included for an independent analysis (beyond the scope of this article) involving repetition suppression analysis.

**Figure 2:**
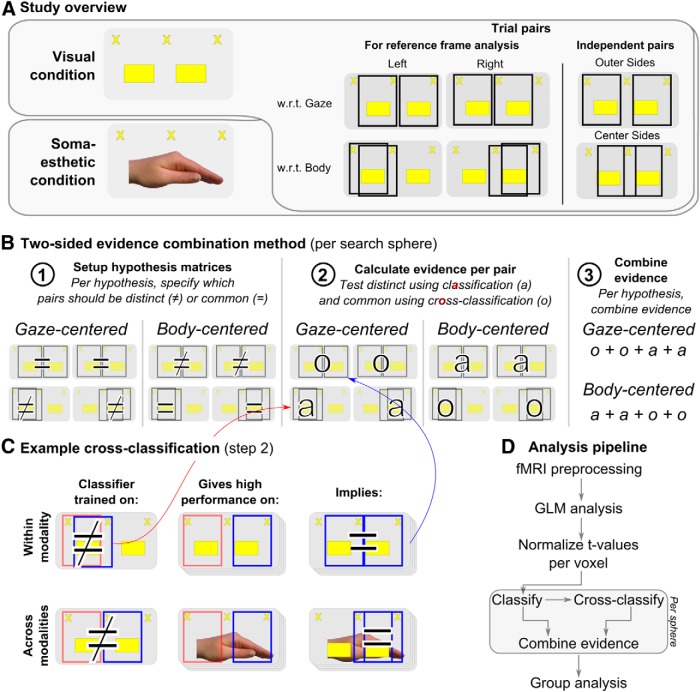
Experimental and methodological framework. ***A***, Study rationale. We studied reference frames for planning grasping movements to targets provided in two modalities: visual (top) or somaesthetic (bottom). Within each modality, reference frames were tested by aggregating evidence across four pairs of trials, either equal or distinct in gaze-centered or body-centered coordinates [right panels, black rectangles indicate pairs of trials (not shown to subject)]. Two pairs, outer sides and center sides, were not used in the reference frame analysis, but were used to independently define regions of interest and test for gaze-direction effects. ***B***, The two-sided evidence combination method. We specified which pairs of trials (indicated by column headers) should be distinct (≠) or common (=) in GC and BC reference frames (1). Next, we calculated the classification score (a) for each distinct pair and the cross-classification score (o) for each common pair (2), and combined the values to obtain an evidence score per reference frame (3). ***C***, Example classification and cross-classification procedure, within (top) and across (bottom) modalities. For each pair of trials (e.g., left w.r.t. body), a classifier was trained and cross-validated (top, first column). The resulting accuracy value represents the classification result (≠ or a) for the specific pair. Next, the trained classifier was tested on the four possible pairs with one of the two trial configurations replaced (e.g., gaze center**-**target left replaced by gaze right**-**target right, second column). In the example, if cross-classification is high (=), it gives evidence for gaze-centered coding, as the pair describing the replacement is equal in gaze-centered coordinates (third column). The procedure was also applied across modalities (bottom), training on one modality (first column) and testing on the other (second column) and vice versa, to test for common representations across modalities (third column). ***D***, Schematic overview of the analysis pipeline, highlighting the most important steps in the analysis.

We focused the experiment on the four combinations of gaze and target position, which can be directly compared between a gaze-centered and body-centered reference frame, as follows: gaze left-target left; gaze center-target left; gaze center-target right; and gaze right-target right (Fig. 2). Configurations with the target further separated from the gaze line (gaze left-target right; gaze right-target left) were not tested, as these trial types had no counterpart in gaze-independent, body-centered coordinates, because in body-centered coordinates the target was always directly to the left or right. Because the head and body were immobilized and the arm had a fixed starting position during the planning phase, head-, hand-, body-, and space-centered reference frames can be treated as equivalent, and were referred to as a body-centered reference frame. Likewise, under the present conditions, retinocentric, eye-centered, and gaze-centered reference frames could be considered synonymous, and were referred to as a gaze-centered reference frame.

Trials were grouped in 16 runs of 22 trials each (on average). Trial order within and across runs was arranged such that each of the four trial types followed each other equally often, while appearing random to the participant (Brooks, 2012). The length of the breaks between runs was determined by the participant. Visual and somaesthetic runs were alternated. The condition (visual or somaesthetic) of the upcoming run was indicated at the end of each break by an auditory signal, which instructed the participant to position the grasping device accordingly. Each run started and ended with 20 and 10 s of fixation, respectively. These intervals served as baseline in the general linear model (GLM) analysis. The total duration of the experiment (with eight runs for each of the two conditions and 88 trials for each gaze–target combination) was 64 min.

### MRI settings and preprocessing

MR images were acquired using a Siemens Tim Trio 3 tesla MRI scanner with an eight-channel head coil. A multiecho sequence of two echoes (TE, 14 and 34 ms; TR, 1.63 s) was used. The sequence encompassed 28 slices, centered on the parietal and frontal motor areas (voxel size, 3.5 mm isotropic; FOV, 192 mm; flip angle, 80º). In addition, high-resolution anatomical images were acquired using T1-weighted MP-RAGE generalized autocalibrating partially parallel acquisition (176 sagittal slices; voxel size, 1 × 1 × 1 mm; TR, 2300 ms; TE, 3.93; FOV, 256 mm; flip angle, 8º). The first echo was used to estimate realignment parameters, as it gives the best estimate of head motion, after which the parameters were copied to the second echo, which was used to estimate the BOLD signal (Poser et al., 2006). Slices were temporally aligned to the center slice (14th) to accommodate for slice-timing differences. High-pass filtering (cutoff, 128 s) was applied to filter out low-frequency confounds. To retain maximal pattern information, no spatial smoothing was applied. Functional data were normalized to MNI space using the DARTEL normalization procedure (Ashburner, 2007), retaining the original dimensions of the data. To estimate the normalization of the flow fields, the structural images were segmented by tissue type. A high-resolution MNI152 template (Fonov et al., 2011) was used to reconstruct and inflate the cortical sheet, separately for the left and right hemisphere, using the FreeSurfer Toolbox (Dale et al., 1999; Fischl et al., 1999). All further processing and analysis steps were performed using SPM8 [Statistical Parametric Mapping (http://www.fil.ion.ucl.ac.uk/spm/)] and Matlab (MathWorks).

### fMRI analysis

As the basis of the analysis, a GLM was run to estimate the number of responses per voxel, after which the searchlight-based evidence combination method was applied, combining classification and cross-classification measures (Fig. 2*D*).

#### GLM

In each of the 16 runs, there were four regressors of interest (i.e., square waves encompassing the delay period). These regressors captured variance related to planning movements in the four tested spatial configurations (gaze left-target left, gaze center-target left, gaze center-target right, and gaze right-target right). The four hemodynamic regressors of interest were convolved with a standard hemodynamic response function (Friston et al., 2011).

Additional regressors were used to constrain the variance explained by the planning regressors. First, we included seven hemodynamic regressors. Four of those seven regressors were square waves encompassing the movement period, from presentation of the Go cue until the return of the hand on the home key (for details, see Experimental setup). These were used to capture variance related to movement execution separately for each of the four trial configurations. Two other regressors were spikes, time locked with stimulus presentation and with the saccade cues. These captured transient stimulus- and saccade-specific effects. A seventh spike regressor, time locked at the onset of a run, accounted for transient effects related to task onset. The seven regressors of noninterest were convolved with the same standard hemodynamic response function as the regressors of interest. Second, we included 17 nuisance regressors. Twelve movement regressors (translation and rotation, as well as their derivatives) captured signal variance caused by head movements. Five additional regressors accounted for the variability in overall image intensity in five compartments that are not expected to hold task-related activity (white matter, cerebrospinal fluid, skull, fat, and out of brain; Verhagen et al., 2008).

Runs were modeled separately in the design matrix. Each run contained 28 regressors and, on average 149 scans, resulting in an average of 2383 scans in total. We used the *t* values of the contrast between planning regressors and baseline as the basis for the evidence aggregation analysis, which is described below. We chose *t* values over β values because *t* values have been shown to provide more information in classification analyses (Misaki et al., 2010).

#### Searchlight analysis

All analyses were performed within local searchlight spheres (Kriegeskorte et al., 2006) with a radius of two voxels (7 mm), moved across the cortex. On average, the sphere size was 30 voxels (or 1286 mm^3^), with smaller search spheres at the outer cortical borders. Instead of ascribing the classification values to the center voxel of a sphere, we averaged, for each voxel, all classification results of the spheres containing the particular voxel. For example, if a voxel was included in 30 search spheres, the ascribed classification result for that particular voxel was the average of the 30 search spheres. This procedure allows for smooth searchlight maps and a better impression of the contribution of single voxels (Björnsdotter et al., 2011; Etzel et al., 2013).

### Reference frame analysis

#### Rationale

The goal of the reference frame analysis was to distinguish between gaze-centered and body-centered coding of visual and somaesthetic targets for grasping. We detected shifts in the weighting of sensory evidence by comparison of the reference frames in fMRI activation patterns between modalities. Modality-independent coding was tested by comparing the cortical spatial code explicitly for similarity across modalities.

The basis of the analyses were pairs of the four trial types (gaze left-target left, gaze center-target right, gaze center-target left, and gaze right-target right), together defining six unique pairs in relation to the location of the target (Fig. 2*A*,*D*), as follows: left w.r.t. gaze, right w.r.t. gaze, left w.r.t. body, right w.r.t. body, outer sides, and center sides. For the first four pairs (Fig. 2*A*), each reference frame makes a prediction on whether the cortical representation should be distinct or common (Fig. 2*A*). For example, in an area using gaze-centered coding, gaze left–target left should invoke a different pattern of activity than gaze center–target left, as the target is at opposite sides of the gaze. In contrast, body-centered coding predicts those patterns of activity to be similar, because the target is on the same side of the body midline (thus, the pair is called the left w.r.t. body). The latter two pairs (Fig. 2*A*, right side) do not lead to reference frame-specific predictions and are instead used to define regions of interest (ROIs).

While previous analytical approaches have focused either on distinct (Beurze et al., 2010) or on common representations (e.g. repetition–suppression analyses; Bernier and Grafton, 2010), here we combine the evidence provided by distinct and common representations. We do so by using searchlight-based classification and cross-classification, aggregating the respective accuracies into an evidence score per the hypothesis. That is, we regard the outcome of classification tests, often referred to as information (Kriegeskorte et al., 2006), as evidence for a particular hypothesis or reference frame. The method first specifies, for the reference frame tested, whether the representations for each pair of trials should be common or distinct (Fig. 2*B*, step 1). Next, per search sphere, classification accuracy is calculated when a distinct representations is predicted, and cross-classification accuracy when a common representation is predicted (Fig. 2*B*, step 2). Finally, we combined the evidence from classification and cross-classification for either a body-centered or a gaze-centered reference frame (Fig. 2*B*, step 3). We will now explain the details of the procedure (Fig. 2*C*).

#### Classification and cross-classification

Classification analysis tests for differences between representations (Cox and Savoy, 2003; Kriegeskorte and Bandettini, 2007); more dissimilar representations show higher classification. Classification was performed separately per the modality, that is, across 8 of the 16 runs. Before classification, we *z*-scored the *t* values per voxel (Misaki et al., 2010). Then, for each pair of trial configurations (six in total, as shown in Fig. 2*A*), we trained and tested a binary linear support vector machine classifier [as implemented in Donders Machine Learning toolbox (https://github.com/distrep/DMLT)]. Leave-one-run-out cross-validation was applied to avoid overfitting of the data. Each run contained four patterns (i.e., the *t* values for the four planning regressors), corresponding to the four trial configurations. Per binary distinction, two of four patterns were used for each of the eight runs, generating 16 patterns. For each cross-validation fold, the classifier was trained on seven of the eight runs, or 14 patterns, and tested on the 2 patterns of the remaining run. The average classification performance across folds constituted the classification score for one pair, that is, the evidence about the dissimilarity of two representations.

Cross-classification analysis tests whether representations are similar (Etzel et al., 2008; Zhang et al., 2013; also called generalization analysis, see Barany et al., 2014); higher cross-classification scores indicate more similar representations. The per-sphere cross-classification scores were based on tests of the six trained binary classifiers (trained as described in the previous paragraph) on the two nontrained conditions. For each cross-validation fold, the tested conditions were taken from the runs not actually trained on. Critically, as the cross-classification analysis tests for generalization, only pairs not used to train the original classifier received a cross-classification score in this way.

For example, consider a classifier trained on the left w.r.t. body pair (i.e., gaze left-target left vs gaze center-target left; Fig. 2*C*, top, first column). For cross-classification, the trained classifier is tested on a comparable distinction, but with one of the two trial configurations replaced (gaze center-target left is replaced by gaze right-target right (second column). Critically, this replacement is different in body-centered coordinates (from left to right), but is equal in gaze-centered coordinates (still left from gaze). Hence, if the cross-classification returns high performance, this can be taken as evidence for gaze-centered coding. The cross-classification evidence is ascribed to the pair describing the replacement, left w.r.t. gaze (third column). Such a replacement can be made in four ways, hence four pairs receive cross-classification evidence from one classification pair. After cross-classification was performed for all six binary classifiers, the resulting cross-classification scores were averaged per receiving pair. This average cross-classification score indicates the similarity of a pair of representations.

#### Combination of evidence

The classification and cross-classification scores constitute separate evidence for the two reference frames, which were combined into evidence measures per reference frame. The rationale for combining the two measures is that classification and cross-classification are complementary measures, giving evidence on two separate sides of a hypothesis: which representations are predicted to be distinct, and which are predicted to be equal. In methodological terms, the measures together disambiguate chance-level performance on either measure. For example, a classification score can be at chance level for the following two reasons: either patterns are too noisy/inconsistent or patterns are equal. In the former case, cross-classification scores will be low; in the latter, cross-classification scores will be high. In addition, when an area shows a high cross-classification score for a pair of patterns, a classifier specifically trained on the distinction between the two seemingly similar patterns could still be able to distinguish them. This would mean the patterns share particular characteristics, but differ in other aspects, allowing the detection of overlapping distinct and common representations. Last, a region could show neither a classification nor a cross-classification effect when no consistent signal is present or two effects in opposite directions conflict.

**Figure 3:**
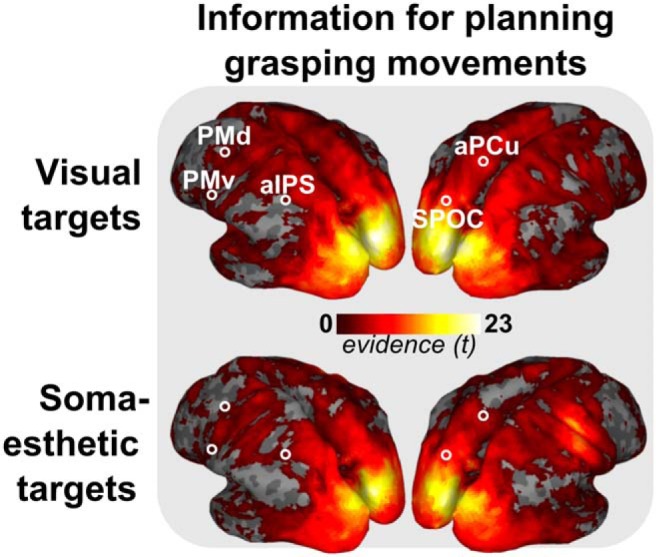
Group *t* values representing information for planning grasping movements, based on classification across trials that differ in both gaze- and body-centered frames of reference (center and outer sides pairs; see Fig. 2*A*, right side), in the visual (top) and somaesthetic (bottom) conditions. Results are shown on an inflated representation of the cortical surface. Color code shows *t* values for consistent information on the distinction between the items of both pairs (*p* < 0.05, uncorrected; cluster size, >250). Peaks in *t* values in either modality served as the basis for the ROI definition.

**Figure 4:**
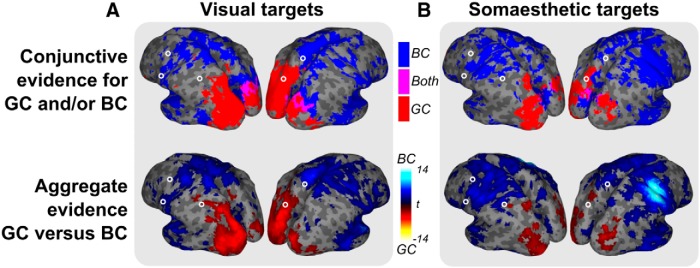
Evidence maps for reference frames (GC and BC) per modality (***A***, visual targets; ***B***, somaesthetic targets) obtained either by the conjunction of classification and cross-classification evidence for each reference frame (top row) or by contrasting the aggregate evidence between reference frames (bottom row). The top row shows binary maps for body-centered (blue), gaze-centered (red), and both reference frames (magenta). The bottom row shows *t* values for the difference in aggregate values between gaze-centered and body-centered evidence (cold colors, BC; warm colors, GC). Note the consistent division between posterior GC and anterior BC evidence in both modalities, across panels. Only relevant clusters are included (*p* < 0.05, uncorrected; cluster-size, >250); open circles indicate ROI locations.

**Figure 5:**
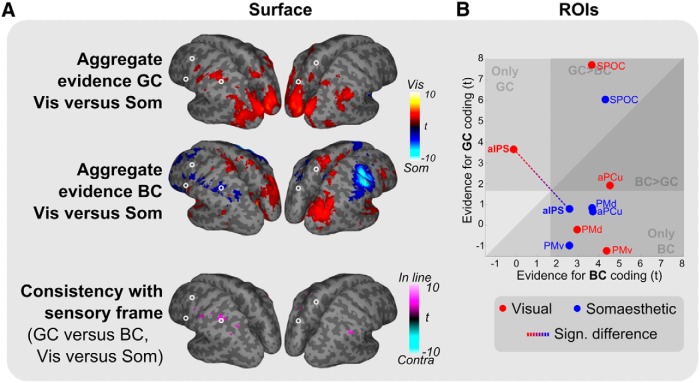
Differences in reference frame-specific tuning between modalities (GC and BC). ***A***, Comparison of GC (top), BC (middle), and GC versus BC (bottom) tuning between modalities. For GC and BC, warmer and cooler colors indicate reference frame-specific dominance in the visual (Vis) and somaesthetic (Som) conditions, respectively. For the consistency plot (bottom, GC vs BC and Vis vs Som, which is the contrast between the GC and BC effects shown in the top two panels, showing only voxels significant in both top panels), magenta indicates a reference frame shift consistent with the sensory reference frame (In line), cyan inconsistent (Contra, not present). Only relevant clusters are shown (*p* < 0.05, uncorrected; cluster size, >250). ***B***, Scatter plots plotting GC versus BC coding per ROI. Colors indicate the two sensory conditions (red, visual; blue, somaesthetic). Dashed line highlights the significant reference frame shift in aIPS in GC, BC, and GC versus BC coding.

**Figure 6:**
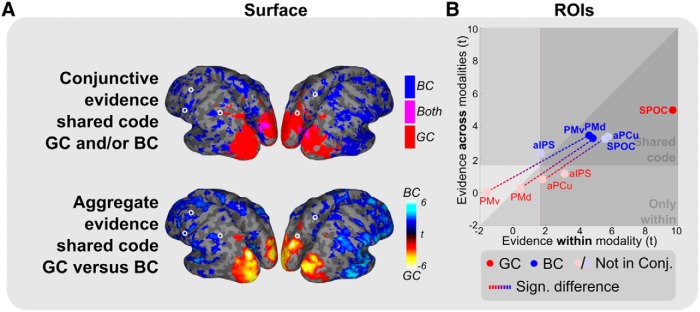
Modality-independent reference frames (GC and BC). ***A***, Binary maps based on the conjunction analysis (top: blue, BC; red, GC; magenta, both) and the difference in aggregate evidence between the reference frames (bottom: cool colors, BC; warm colors, GC). Only relevant clusters are shown (*p* < 0.05, uncorrected; cluster size >250). Occipitoparietal cortex shows a modality-independent gaze-centered reference frame. A shared body-centered reference frame is seen in part of the frontal regions. Circles indicate ROIs. ***B***, Comparison of *t* values for within-modality coding (sum of visual and somaesthetic, *x*-axis) versus across-modality coding (sum of both directions of cross-classification, *y*-axis) for the two reference frames (red and blue dots; transparency indicates that conjunction results are not significant for the respective reference frame and ROI, as shown in ***A***, top). Dashed lines highlight significant differences between reference frames (*p* < 0.05). Areas highlighted by the triangular dark zone, marked as “Shared code,” show evidence for a shared, modality-independent coding for the indicated reference frame.

We combined the evidence across pairs using two approaches. First, we considered whether both sides of the evidence (classification and cross-classification) were in line with the hypothesized reference frame. For gaze-centered coding, left or right w.r.t. gaze cross-classification and left w.r.t. or right w.r.t. body classification needed to be significant (for details on significance, see Group analysis). For body-centered coding, a left or right w.r.t. gaze classification and a left or right w.r.t. body cross-classification were required to be significant. This combination of significant evidence in classification and cross-classification analyses was labelled as conjunction. It allows for detecting the presence of either or both reference frames, leading to binary maps (see Figs. 4, top row, 6, top row, 7). Second, we considered whether the average across classification and cross-classification scores were in line with the hypothesized reference frame: for gaze-centered coding, the cross-classification values for left and right w.r.t. gaze and the classification values for left and right w.r.t. body. For body-centered coding, we averaged the classification values for left and right w.r.t. gaze, and the cross-classification values for left and right w.r.t. body. This combination of evidence was labeled as aggregation, and is depicted as scalar values on surface maps (Figs. 3, 4, bottom row, 5, 6, bottom row, 7) and in ROI-specific measures (Figs. 5*B*, 6*B*). It allows for detecting relative dominance in the reference frame with high sensitivity, but low specificity. Note that both gaze-centered and body-centered measures have an equal number of classification and cross-classification scores, which cancels out any possible imbalance between classification and the more stringent cross-classification scores.

To illustrate the low sensitivity of the aggregation method, body-centered coding predicts high classification on two of the four main pairs and high cross-classification on two other pairs (Fig. 2*B*). Critically, the two classification pairs differ not only in body-centered coordinates, but also in the direction of the gaze (Fig. 2*A*, compare the left and right w.r.t. bodies). This means that gaze-direction coding also predicts high classification scores for the two pairs, and thus a high average score. In other words, using the aggregate measure, body-centered coding is confounded by gaze-direction coding (as is gaze-centered coding; compare left and right w.r.t. gaze). The conjunction analysis, however, also explicitly requires the two cross-classification pairs to be significant, which are not predicted to be significant for gaze direction, separating the two explanations.

Contrasts between aggregate evidence values for the reference frames are unaffected by gaze direction, as an equal number of classification and cross-classification scores are included on both sides, such that the gaze-direction effects cancel out. Rather, conjunction and contrasts between aggregate values, as used in the overlay images, are likely to give similar results, as the reference frames are the mirror images of each other (Fig. 2*B*), and the classification and cross-classification for a pair are, on average, negatively correlated.

As an additional control measure, we explicitly tested for gaze-direction effects by testing for significant classification on all four main pairs, combined with significant cross-classification on the center-sides pair. The latter pair is the only pair predicted to be common in regions coding gaze direction, but distinct in either reference frame, allowing direct estimation of a possible gaze-direction effect.

#### Within- versus across-modality coding

We tested both the dominant reference frame within a modality and the consistency of coding across modalities; that is, the grasp areas that determine reference frames depending on the sensory modality, and those that code representations using modality-independent codes.

To test reference frame evidence within a modality, both training and test data were from the same modality (Fig. 2*C*, left). To test reference frame-specific common coding across modalities, the training and test data were from different modalities (Fig. 2*C*, right), averaging the two directions (from visual to somaesthetic and vice versa). Thus, for across-modality coding, we applied cross-modal classification (Etzel et al., 2008).

### Group analysis

For group analysis, we ran a *t* test on the evidence maps for gaze-centered, body-centered, and gaze-direction coding across subjects. The GLM was implemented in the GLM Flex toolbox (http://mrtools.mgh.harvard.edu/index.php/Main_Page). All group *t* tests were run using a GLM with 432 scans (18 subjects × 2 modalities × 6 trial pairs × 2 tests) and 42 regressors (18 subjects + 2 modalities × 6 trial pairs × 2 tests), or a df of 432 − 41 = 391. For surface results, results were thresholded at α = 0.05, cluster size >250 (α, ∼ 0.1, cluster-level corrected), to display the whole range of effects in the data. Moreover, full cluster correction on average searchlight results has proven to be conservative (Stelzer et al., 2013).

### Regions of interest

Because we had clear hypotheses on the cortical pathways involved in planning right-handed grasping movements, we focused our analyses on five ROIs in the left hemisphere. These ROIs characterized areas along the dorsolateral and dorsomedial pathways that have been implicated in reach and grasp control (Connolly et al., 2003; Verhagen et al., 2008; Filimon et al., 2009; Bernier and Grafton, 2010; Fabbri et al., 2014). Each ROI was determined on the basis of the local maxima in evidence for the independent pairs (center sides and outer sides; Fig. 2*A*; Kriegeskorte et al., 2009) closest to the average coordinates reported in the study by Bernier and Grafton (2010). The local maximum was determined on the basis of the group *t* values for the combined classification score for the two pairs, as these two pairs are predicted to be distinct in both reference frames. The peak could be in visual coding, somaesthetic coding, or in the sum of their scores. Peaks were restricted to the cortical surface, and the ROI was defined as a 7-mm-radius sphere surrounding the peak.

Along the dorsolateral pathway, we considered anterior intraparietal sulcus (aIPS) and ventral premotor cortex (PMv). The area of the aIPS [reference, −35, −47, 50; centered at, −32, −42, 46 (*x*, *y*, *z* in MNI coordinates)] has been shown to be involved in planning grasping movements (Grafton et al., 1996; Binkofski et al., 1998; Culham et al., 2003; Dinstein et al., 2008; Verhagen et al., 2008). PMv (reference, −52, −1, 31; centered at, −56, −3, 32) has been implicated in movement preparation, including the preshaping of the hand during grasping (Toni et al., 2001; Davare et al., 2006; Hoshi and Tanji, 2007).

Along the dorsomedial pathway, we selected the superior parieto-occipital cortex (SPOC), anterior precuneus (aPCu), and dorsal premotor cortex (PMd). The SPOC region (reference, −16, −77, 40; centered at, −17, −74, 46) has been implicated in reaching and grasping movements (Connolly et al., 2003; Culham and Valyear, 2006; Verhagen et al., 2008; Bernier and Grafton, 2010). As a region, SPOC has also been referred to as parieto-occipital junction (Prado et al., 2005; Bernier and Grafton, 2010) and as the human homolog of macaque V6A (Pitzalis et al., 2013). The aPCu region (reference, −9, −55, 63; centered at, −4, −56, 63) has been involved in reaching movements, independent of visual feedback (Filimon et al., 2009; Wenderoth et al., 2006), using a reference frame that is dependent on the sensory modality of the target (Grafton, 2010). This region falls into the probability maps of Brodmann areas 7A and 5L (Eickhoff et al, 2005). Dorsal premotor cortex (reference, −21, −10, 56; centered at, −28, −4, 56) has been involved in planning and controlling both reaching and grasping (Davare et al., 2006; Raos et al., 2006; Hoshi and Tanji, 2007).

## Results

We investigated the reference frames in which parietal and frontal regions operate during the planning of grasping movements toward visual and somaesthetic targets. In the experiment, we manipulated the position of a grasping target (left or right from the body midline) and the direction of gaze (left, center, right). We focused our analyses on four pairs of trials (left or right w.r.t. gaze or body; Fig. 2*A*), which could either be different or equal in GC and/or BC coordinates. We used a novel searchlight-based evidence combination method to take into account the following two types of evidence: classification tested for distinct representations (i.e., where coordinates are predicted to be different in the tested reference frame); and cross-classification for common representations (i.e., where coordinates are predicted to be equal; for details, see Materials and Methods; Fig. 2). Based on the specific predictions of each reference frame, the two pieces of evidence were combined into an evidence score. We used comparisons between the modalities and cross-classification across modalities to test which regions code a representation linked to the reference frame of the sensory modality and which code a common, modality-independent representation.

Behavorial analysis showed that participants performed the task effectively, with virtually no errors in target selection (0.05% error; range, 0–3.05% across subjects), and matched performance across modalities, both in reaction time (reaction time to visual targets: 630 ms; SD, 189 ms; reaction time to somaesthetic targets: 626 ms; SD, 181; *t*_(34)_ = −0.07, *p* > 0.05) and movement time (movement time to visual targets: 2.465 s; SD, 504 ms; movement time to somaesthetic targets: 2.574 s; SD, 551 ms; *t*_(34)_ = 0.62, *p* > 0.05).

In the following, we start by describing the cortical distribution of planning-related information that is distinct in both gaze- and body-centered reference frames. This information, which is based on the classification of center-sides and outer-sides pairs (Fig. 2*A*, right side) served as the basis for the independent definition of the ROIs. Next, we use the remaining trial types to decode the reference frames involved in processing target information in visual and somaesthetic modalities, followed by a comparison between modalities and an examination of regions that use modality-independent codes. We end with an analysis of the cortical topography of gaze direction, based on cross-classification of the center-sides pair (i.e. trials with identical gaze position).

### Definition of ROIs

To allow for independent ROI definition, we tested for information on grasp planning to the two targets with gaze either at the center or on the outer sides of the target (Fig. 2*A*, right side), because in this case the target is distinct in both gaze- and body-centered reference frames. Figure 3 shows that the cortical distribution of the classification information is similar when the target was presented visually or somaesthetically. The indicated ROIs were defined based on the evidence peaks in either modality.

### Reference frames per modality

Figure 4 shows the reference frame scores across the surface, which were based on the combination of evidence across four of the six pairs of trial types, separately for each reference frame and modality.

Planning grasping movements to visual targets evoked gaze-centered representations only in occipital and parietal cortices (Fig. 4*A*, top). Body-centered coding was present in the left frontal and right parietofrontal regions. The contrast between references frames values confirmed that tuning was predominantly gaze centered in occipitoparietal cortex and predominantly body-centered in frontal regions (Fig. 4*A*, bottom).

For somaesthetic targets, we found gaze-centered information in bilateral occipitoparietal cortex only, as for visual targets (Fig. 4*B*, top). Body-centered information was present in frontal regions, extending into bilateral rostral parietal cortex. The contrast between the reference frames showed that gaze-centered tuning was dominant in small parts of occipital and parietal cortex, while more frontal regions, and rostral parietal cortex, were biased to body-centered coordinates (Fig. 4*B*, bottom). The strongest peak was found in right somatosensory and motor cortex, probably reflecting the representation of the somaesthetic targets, provided by the left hand.

The predefined ROIs further characterize these visual and somaesthetic grasping gradients, with some predominantly gaze-centered (SPOC), others predominantly body-centered (aPCu and the frontal ROIs). The area aIPS appears to change its dominant reference frame according to the sensory input modality. This effect is further explored below.

### Modality-dependent reference frames

We further characterized how the relative contribution of these two reference frames depends on input modality, by examining the differences between modalities. Figure 5 shows the results of this analysis.

Gaze-centered tuning was stronger for visual targets in a large caudal bilateral cluster ranging from the occipital cortex, along the left IPS, to aIPS at the junction with postcentral sulcus (Fig. 5*A*, top row). No regions showed a bias for gaze-centered tuning for the processing of somaesthetic targets, relative to visual targets. Body-centered coding was dominant for somaesthetic targets in right S1 and M1, probably reflecting information on the left hand (which provides the somaesthetic targets). Importantly, in the dorsolateral parietofrontal pathway, the left aIPS also showed body-centered dominance for somaesthetic targets (Fig. 5*A*, middle row). The consistency analysis (Fig. 5*A*, bottom row) showed a clear switch, consistent with the sensory reference frame, specifically in left aIPS.

ROI analysis confirmed the findings of the whole-brain analysis (Fig. 5*B*). Of the ROIs, only left aIPS showed significantly higher gaze-centered tuning in the visual condition (*p* < 0.05), significantly higher body-centered tuning in the somaesthetic condition (*p* < 0.05), as well as a significant difference in relative tuning (GC-BC) between the visual and somaesthetic conditions (*p* < 0.005). Other ROIs did not show significant evidence for a switch in reference frame across modalities.

Thus, aIPS was the only region in which it was possible to identify a modality-dependent reference frame for grasp planning.

### Modality-independent reference frames

We next test whether the similarities in reference coding between modalities (Fig. 4) also imply modality-independent cortical coding (Fig. 6); that is, whether the same pattern in fMRI activations is used to represent the targets in a particular reference frame, irrespective of target modality.

Results showed extensive modality-independent coding (Fig. 6*A*). The gaze-centered representations in bilateral occipitoparietal cortex as well as part of the body-centered representations in frontal regions were shared across modalities.

The ROIs (Fig. 6*B*) show a strong division between body- and gaze-centered codes. Body-centered coding is shared across modalities in almost all ROIs (all BC, *p* < 0.01; significant conjunctive evidence in PMv and PMd). Only in aPCu, PMd, and PMv, the strength of common body-centered coding was significantly higher than that of common gaze-centered coding (GC-BC, *p* < 0.05). In contrast, gaze-centered coding is specifically shared across modalities in SPOC (GC, *p* < 0.001, no difference between GC and BC). Importantly, even though SPOC appears to code both reference frames using a multimodal code when tested using the aggregate measure in the ROIs (Fig. 6*B*), only the gaze-centered coding is significant in the conjunction test (Fig. 6*A*,B, compare transparent dot, opaque dot).

In sum, areas PMd, PMv, and aPCu contain a modality-independent, body-centered code while area SPOC comprises a gaze-centered modality-independent code during grasp planning.

### Effect of gaze direction

**Figure 7:**
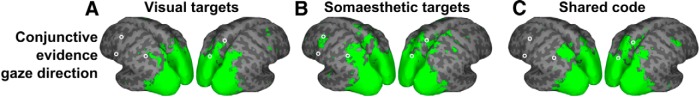
***A–C***, Gaze-direction coding for planning grasping movements to visual targets (***A***), somaesthetic targets (***B***), and irrespective of modality (***C***). Voxels within the binary map are significant for classification on all four main pairs and cross-classification on the center-sides pair, as determined using the conjunction test. Compare to Figure 4*A* (top row) for an overview of all tuning. Circles indicate ROIs.

Finally, because there may be gaze-direction effects in the data (for details, see Materials and Methods), we specifically tested for such effects in Figure 7.

Gaze-direction coding was found primarily in the occipital cortex, extending into the parietal cortex (Fig. 7*A*,B), irrespective of modality (Fig. 7*C*). Note the striking overlap between gaze information and gaze-centered coding in Figures 4 and 6, meaning gaze information and gaze-centered coding occupy similar regions. Of the predefined ROIs, we found that only SPOC coded gaze direction consistently within modalities (both *p* < 0.01, no difference between the visual and somaesthetic conditions) and across modalities (*p* < 0.05). The latter could also explain the discrepancy between the body-centered tuning in SPOC when using the aggregate measure (Figs. 5*B*, 6*B*) and the absence of such tuning in the conjunction measure (Figs. 4, 6*A*, top row). Note that the conjunctive measure and the gaze-direction measure are independent, reaffirming the conclusion that gaze-direction and gaze-centered tuning are present in similar areas.

## Discussion

This study investigated the reference frames used to plan grasping movements toward visual and somaesthetic targets. We focused on the manifestations of gaze- and body-centered frames of reference in the dorsomedial and dorsolateral grasping-related circuits. There are three main observations. First, a parietal node of the dorsolateral circuit (aIPS) encoded the grasping target flexibly, in either a body-centered or a gaze-centered reference frame, depending on whether the movement was planned toward a somaesthetic or a visual target. Second, posterior regions, including SPOC, coded the grasping target in gaze-centered coordinates, while anterior regions, including PMd and PMv, operated in a body-centered reference frame. Third, gaze- and body-centered coding of the grasping target was largely modality independent. These observations are based on a novel analysis method, combining multivariate classification and cross-classification evidence. Each of these points will now be discussed in detail.

### Flexible reference frame in aIPS

In the dorsolateral circuit, we find a modality-dependent reference frame in aIPS, which switches between gaze and body coordinates according to the sensory modality used to spatially define the grasping target. This observation is consistent with recent ideas predicting reference frame switches alongside the native reference frame of the sensory signal (Pouget and Snyder, 2000; McGuire and Sabes, 2009). For visual targets, input is coded with respect to gaze; for somaesthetic targets, input is coded with respect to the body. Thus, aIPS appears to code targets in a statistically optimal fashion, favoring coding in the presented sensory reference frame (Pouget et al., 2002).

Area aIPS is well positioned to play such an integrative role in planning grasping movements, both in functional profile and connectivity. Functionally, aIPS is necessary for appropriate planning of grasping movements (Gallese et al., 1994; Binkofski et al., 1998; Culham et al., 2003; Verhagen et al., 2012), and it represents a wide range of target properties across modalities (Murata et al., 1996; Grefkes et al., 2002). Anatomically, the area is densely connected to both visual, somatosensory, and premotor areas (Lewis and Van Essen, 2000; Nakamura et al., 2001; Borra et al., 2008), allowing it to integrate the information required for representing the properties of grasping targets, and switch reference frame based on sensory conditions. In line with our results, neurons in the homolog area in the macaque use predominantly gaze-centered representations when coding grasping movements to visual targets (Lehmann and Scherberger, 2013). This could imply that the current results generalize from the population level to the neuronal level. However, the relatively more anterior gaze-centered zone and the relatively more posterior body-centered zone could also point to separate functional populations within the aIPS area (Romero et al., 2014). Single-neuron studies for grasping movements to somaesthetic targets, combined with repetition–suppression analyses of the current dataset, might be able to address this issue.

In the dorsomedial circuit, we find no evidence for a switch between reference frames. Recently, Bernier and Grafton (2010) found that the dorsomedial region aPCu codes a modality-dependent switch in reference frame for reaching movements with the index finger, which is analogous to the effect found in aIPS in the present grasping study. There are a number of possibilities accounting for this difference. First, it is possible that the current experimental design, focused on the planning phase of a grasping movement, is not optimal for detecting effects that might occur predominantly during the execution of the movement. It has been shown that the dorsolateral pathway, including aIPS, is involved earlier in planning a grasping movement than the dorsomedial pathway, including SPOC and aPCu, which is only involved just prior to the movement (Verhagen et al., 2012, 2013; Lehmann and Scherberger, 2013). Although the design of this study ensures that the findings are not influenced by stimulus processing, movement execution, or somatosensory reafference following the movement, sensitivity might have been biased toward planning-related processes supported by the dorsolateral pathway. Second, the nature of the sensorimotor transformations required by the task settings might also play a role. In this study, visual and somatosensory frames of references were in register, given that participants had direct line of sight to fixation lights and grasping targets. In other studies, there was a substantial difference between the line of gaze and the somatosensory location of the reaching target (Bernier and Grafton, 2010). It is conceivable that the dorsomedial stream might become particularly relevant when a discordance between visual and somatosensory frames of references is corrected (Luauté et al., 2009). Third, from the perspective of an optimal control framework (Todorov and Jordan, 2002; Scott, 2004), the use of a flexible reference frame might be linked to the task-relevant effector. In the current study, the end-effector is given by thumb and index fingers articulating over the wrist (Fig. 1). In contrast, Bernier and Grafton (2010) used the whole forearm, from elbow to index finger, as a single effector. It has been shown that there is a cortical dissociation between hand and finger movements, represented in aIPS, and whole-limb movements, represented in aPCu (Heed et al., 2011; Leoné et al., 2014; Sereno and Huang, 2014) and other areas of the dorsomedial circuit (Cavina-Pratesi et al., 2010a,b). Accordingly, switching between gaze- and body-centered reference frames might be implemented in the parietal region, which is more directly involved in representing the combination of limb segments controlled during task performance. For example, reference frame switches for foot/leg movements are predicted to peak in more medial regions than reported here (Leoné et al., 2014).

### Gaze- versus body-centered networks

The present study indicates that areas aIPS and aPCu fall between posterior gaze-centered coding and anterior body-centered responses. Specifically, occipitoparietal cortex, including SPOC, uses a predominantly gaze-centered code for both visual and somaesthetic targets, which is compatible with previous findings on a gaze-centered dominance in parietal cortex (Stricanne et al., 1996; Crawford et al., 2011), and the visual and somaesthetic inputs reported in these regions (Fattori and Gamberini, 2001; Fattori et al., 2005, 2009). In addition to these gaze-centered codes, we found evidence for gaze-direction coding in parietal regions (Galletti et al., 1995; Marzocchi et al., 2008; Hadjidimitrakis et al., 2011; Rossit et al., 2013). Access to this information may allow the regions to play a role in reference frame transformations (Zipser and Andersen, 1988; DeSouza et al., 2000; Barany et al., 2014). In fact, we believe that a caudal–rostral gaze-centered/body-centered gradient could be underlying our results for posterior parietal cortex (PPC; McGuire and Sabes, 2011). In frontal regions, including areas PMd and PMv, we instead found evidence for body-centered coding, which is in line with the assumed role of premotor regions in motor preparation (Graziano and Gross, 1998) and the implementation of joint-based motor commands (Beurze et al., 2010).

The present results could be taken as inconsistent with other reports showing mixed coding in parietal and frontal regions (Galletti et al., 2003; Avillac et al., 2005; Mullette-Gillman et al., 2005; Pesaran et al., 2006; Batista et al., 2007; Chang and Snyder, 2010), as well as important theoretical work (Pouget and Snyder, 2000). However, caution with such an interpretation is needed. Differently from previous reports, this study did not manipulate the body-centered reference frame by using a different start position for the right hand, leaving open the possibility that gaze-centered responses also become consistent with body-centered coding when both reference frames are manipulated. In addition, dominant gaze-centered coding could obscure body-centered coding and vice versa, as the expected effects in cortical patterns are opposite. Last, gaze-direction effects in parietal regions might have obscured body-centered effects or induced apparent body-centered effects in the current and other studies. For example, in our study SPOC appears to also code body-centered coordinates; however, the conjunction and gaze-direction analysis shows that this is probably an effect of gaze direction.

### Modality-independent reference frames

We found the coding in both the gaze- and body-centered networks to be largely modality independent. This observation extends previous work on modality-independent spatial tuning (McGuire and Sabes, 2011), and on multisensory integration in both parietal and premotor regions (Bremmer et al., 2002; Macaluso et al., 2003; Todorov, 2007) by showing that the coding is not only in the same region, but shows the same within-region cortical pattern (as tested by cross-modal classification; Etzel et al., 2008).

The extent of the modality-independent coding is probably linked to the generality of the function served by the regions. PPC is also believed to code a general saliency map, which generalizes across tasks (Jerde et al., 2012). Within this notion, we show that it also generalizes across modalities. Such modality independence would fit the preserved role of PPC in the congenitally blind (Lingnau et al., 2012). The modality-independent, body-centered code in premotor regions fits the coding of the impending action, which is equal across the two modalities and is believed to be coded in body-centered coordinates. The widespread modality-independent tuning could have been influenced by the small number of sides (two per reference frame); further studies should include more spatial locations to further specify the modality-independent coding. Moreover, other studies should manipulate the properties of the grasping target to examine whether these properties influence the modality dependence (or independence) of tuning.

### Two-sided evidence combination

We introduced a two-sided evidence combination method. This method allows one to combine evidence from both predicted distinctions between cortical representations, using classification, and predicted common representations, using cross-classification. This methodological approach provides access to overlapping representations in gaze- and body-centered frames. Previous methods, which were focused on classification effects or univariate differences, would not have detected common representations. Similarly, repetition suppression approaches would not be able to detect distinct representations.

We used the following two implementations of the method: one qualitative, aimed at explicitly determining regions that are significant for a conjunction of both sides of the evidence; and one quantitative, aimed at differences in the degree of evidence by means of averaging. The method can be further extended to accommodate more complex hypotheses by using GLMs to fit evidence scores rather than calculating averages. The method also combines naturally with the pattern-activation method by Leoné et al. (2014) as well as representational similarity analysis (Kriegeskorte et al., 2008), replacing correlations with cross-classification and classification.

### Conclusion

This study shows that dorsolateral grasping area aIPS switches reference frame depending on the sensory modality: visual targets are predominantly processed in a gaze-centered reference frame, and somaesthetic targets are coded in a body-centered reference frame. In contrast, other parietofrontal regions respond mainly in a single reference frame, with caudal parietal areas in the dorsomedial pathway code grasping targets in a modality-independent, gaze-centered reference frame, while premotor areas code targets in a modality-independent, body-centered reference frame. The modality-independent nature of the parietal and frontal clusters could reflect their roles in coding both saliency and motor preparation. Area aIPS rather serves a potentially fundamental role as an in-between conversion hub when coding grasping movements.

## Figures and Tables

**Figure:**
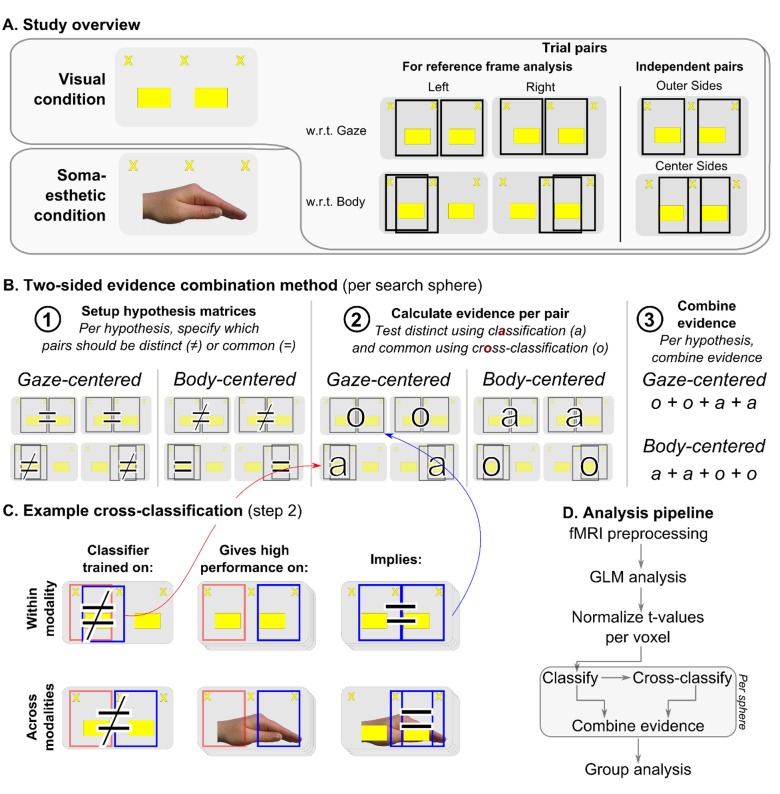


## Synthesis

The decision was a result of the Reviewing Editor Trevor Drew and the peer reviewers coming together and discussing their recommendations until a consensus was reached. A fact-based synthesis statement explaining their decision and outlining what is needed to prepare a revision is listed below. The following reviewer agreed to reveal her identity: Jody Culham

The authors apply a novel and sophisticated multivariate fMRI technique to study the reference frames used by different cortical areas to plan reach-to-grasp actions to both visual and somaesthetic targets. By varying gaze direction and target location, the author wished to test whether the cortical representation of motor goal is gaze-centered and body-centered. In contrast to parietal and premotor regions employing modality-independent coordinates, the authors found that the reference frame of aIPS depends on sensory modality, leading to their main conclusion that aIPS acts as an hub in transition between gaze-centered and body-centered maps. The results are interesting and their interpretation is generally reasonable. However, although the study is well conducted and the results are interesting, both the experimental design and data analysis are complex and require further clarification and justification.

Major comments

1. There are some possible confounding factors in the paradigm design (Fig.1-2, p. 6-7) that are not well-controlled and that need to be seriously discussed. For example, the left hand was involved only in the somaesthetic condition but not in visual condition. In this case, the hand shapes, finger forces, and anticipated sensory consequences might be totally different in two conditions. Why did the authors not add the left hand in the visual condition and present the visual cue with LED attached to the hand? Similarly, why were more target/gaze locations not used to distinguish between eye-centered representation and gain-modulation by gaze direction? The authors should clarify these issues and discuss possible alternative hypotheses.

2: There are several issues concerning the description and presentation of the methodology (pg 8-16) used that need to be addressed. These include:

a) the figures show highly processed data after multiple steps of analyses, including GLM, support vector machine, Z-score transform, etc., the author should re-organize the part to make it clear, and probably add some equations.

b) Pages 7 &amp; 8: The data was subject to a significant amount of normalization (particularly the transformation into MNI space) prior to the searchlight analysis. Some groups using multivoxel pattern analysis recommend keeping the data closer to the native functional space to reduce the number of interpolations or alternatively, if using cortical surfaces, to average within adjacent patches of the surface (but not for example across banks of a sulcus; Oosterhof et al., 2011, NeuroImage). The authors need to discuss their reasoning for using the normalization approach they did?

c) Could the authors clarify that the four main task regressors and seven additional task regressors were square-waves, spikes, and/or convolved with a hemodynamic response function. One assumes so given that it says "hemodynamic regressors" but this should be explicit and the type of hemodynamic model (e.g., two-gamma) should be specified.

d) Page 8: Please clarify further how the first echo was used to estimate realignment parameters.

e) Page 9: Could the authors please clarify the size of the voxels that formed the searchlight sphere. Initially it is confusing to the reader to state that the searchlight sphere has a "radius of two voxels" but a "sphere size" of 30 voxel. In addition, it should be stated explicitly what volume (mm^3) this corresponds to. That is, are the voxels in the native functional resolution (3.5-mm isotropic according to p. 7) or have they been resampled to another resolution during all the normalization? Better wording may be something like, "The searchlight sphere had a radius of two voxels (X mm), such that the volume of the sphere could include up to 30 voxels (X mm^3), with smaller search spheres at the cortical borders." More clarification regarding the "cortical borders" is also needed - could search volumes include voxels on opposite banks of a sulcus?

f) Page 9: Could the authors expand on the sentence "Instead of ascribing the classification values to the center voxel of a sphere, we averaged, for each voxel, all classification results of the spheres containing the particular voxel."

g) Page 11: The wording of the first two sentences of the last (incomplete) paragraph could be clearer about the specific discrimination being trained. Are you training the classifier to distinguish left-vs. right visual field when targets are on the left side of the body and then testing the same discrimination when targets are on the right side of the body?

h) Page 15: Clarify what data was used to select the local maxima in the sentence beginning "Each ROI was determined..." It didn't makes sense.

4: Figure 2 is pretty complicated. Some things that could be removed to increase simplicity would be to delete the "Predictions" in 2A as it contains the same information that is presented in panel 1 of figure 2B. Panels 1 and 2 in figure 2B also contain pretty much the same information. Panel 2 could be removed and ( = and o) replaced with a (y) and (≠ and a) replaced with an (x) Also, the labels at the bottom of figure 2C are not that intuitive. I suggest removing them and clarifying the procedure in the figure caption.

5: Apparently, a score combining classification and cross classification measures was utilized to judge whether the reference frame is closer to gaze- or body-centered. It is interesting to find that aIPS switched between them, but it is oversimplified to claim that other regions indeed are gaze- or body-centered. Throughout the results, discussion, and abstract it could be made clearer that the weighting of gaze- vs. body-centered reference frames is relative rather than absolute. I.e., it is not the case that aIPS ONLY uses an eye-centered reference frame when grasping visual targets, rather aIPS PREFERENTIALLY uses an eye-centered reference frame. The current wording (particularly in the abstract) tends to be stronger (more all-or-none) than the data suggests.

6: The Results section (p. 16-20) could be improved by interpreting results in more coherent way rather than simply listing them individually. Moreover, many relevant parameter are missing. For example, N (trial number) should be given with SD (p. 17 top), N and statistical test should be explicitly stated with p-values (p. 19).

7: Discussion- The present finding that anterior precuneus preferentially uses a body-centered reference frame regardless of target modality is in disagreement with previous findings by Bernier and Grafton (2010) who found that this region could switch between a gaze- and body-centered reference frame depending on target modality. I want to commend the authors for acknowledging this and providing a thorough discussion of potential reasons for this difference.

8: In the Discussion, relevant studies in neurophysiology are absent. Important advances have made in recording from AIP in monkeys (e.g. by Scherberger and colleagues), providing better spatiotemporal resolutions at single-neuron level. This and other work needs to be better discussed and integrated to clarify what new findings the current work brings to our understanding of how reference frames are organised within different structures.

Minor:

There are a number of minor spelling and grammatical errors throughout. I have indicated a few below.

- Abstract, line 10: "... to explicity aggregate evidence in favor (of) and against the presence..."

- Page 6, line 2: "... until both grasping and eye movement(s) were performed..."

- Page 6, line 6: "Trials were only regarded (as) correct when the target..."

- Page 6, 3rd sentence from the end: "... 500 ms after movement o(n)set..."

- Please ensure that all acronyms are defined prior to their first use (i.e, GLM on page 7).

Manuscript Instructions

The species studied is not mentioned in the abstract. Please make sure to update both the abstract in the article file and on the submission form.

We now specify the species in the abstract.

Synthesis of Reviews:

The authors apply a novel and sophisticated multivariate fMRI technique to study the reference frames used by different cortical areas to plan reach-to-grasp actions to both visual and somaesthetic targets. By varying gaze direction and target location, the author wished to test whether the cortical representation of motor goal is gaze-centered and body-centered. In contrast to parietal and premotor regions employing modality-independent coordinates, the authors found that the reference frame of aIPS depends on sensory modality, leading to their main conclusion that aIPS acts as an hub in transition between gaze-centered and body-centered maps. The results are interesting and their interpretation is generally reasonable. However, although the study is well conducted and the results are interesting, both the experimental design and data analysis are complex and require further clarification and justification.

We thank the editor and reviewers for the clear and insightful comments and suggestions. Below we delineate the changes we made and our responses to the integrated review.

Major comments

1. There are some possible confounding factors in the paradigm design (Fig.1-2, p. 6-7) that are not well-controlled and that need to be seriously discussed. For example, the left hand was involved only in the somaesthetic condition but not in visual condition. In this case, the hand shapes, finger forces, and anticipated sensory consequences might be totally different in two conditions. Why did the authors not add the left hand in the visual condition and present the visual cue with LED attached to the hand? Similarly, why were more target/gaze locations not used to distinguish between eye-centered representation and gain-modulation by gaze direction? The authors should clarify these issues and discuss possible alternative hypotheses.

We controlled for differences between the two conditions in the following ways. When practicing the task, participants were instructed to position the left hand in such a way (enforced by the setup) that the right hand grasp was comparable to the grasp in the visual conditions. Moreover, they were instructed to grasp consistently between the fixation lights and with equal force, irrespective of condition. Visual inspection, during practice and during actual execution of the experiment, confirmed their compliance.

We agree there could be a difference in predicted sensory consequences of the grasps, since the two actions are based on different sensory input (visual vs somaesthetic). We did not want to add the left hand in the visual condition and present the visual cue with LED attached to the hand. In our view, it would be flawed to compare a bimodal (visual+somaesthetic) with a unimodal (somaesthetic) condition, as the first evokes convergence mechanisms and the second does not. By using distinct targets for the two conditions, rather than cueing visual cue on top of the hand, we guaranteed there was no direct mapping between the visual and somaesthetic targets. We now note in the text the reason for this choice:

In particular, the visual blocks were brought in front in the visual condition, were illuminated and the left hand was positioned next to the subject on the scanner bed. By keeping the left hand away from the visual blocks and the body midline, we guaranteed there was no effect of left hand position on the visual spatial coding.

Page 5, lines 14-16

We chose the current number of trial configurations (six, of which four were used for the reference frame analysis) as it is the most direct way to test for reference frame effects (see for example also Medendorp et al, 2003; Bernier &amp; Grafton, 2010) and practically doable on the small space of the hand and within reasonable scanning time. After this first test for visual and somaesthetic targets, follow-up studies should ideally use more targets, as we note in the discussion:

The widespread modality-independent tuning could have been influenced by the small number of sides (two per reference frame); further studies should include more spatial locations to further specify the modality-independent coding.

Page 24, lines 9-12

Further, note that the current reference frame results cannot be explained by gain field effects alone. If that would be the case, equal gaze-centered locations with distinct gaze-position (e.g., gaze center-target left and gaze right-target right) would not employ a common representation in gaze-centered coordinates. This is a direct advantage of combining the two sides of evidence. Rather, gaze-dependent gain fields are an effect independent from gaze-centered coding and would reside in the gaze-effects, as shown in figure 7.

2: There are several issues concerning the description and presentation of the methodology (pg 8-16) used that need to be addressed. These include:

a) the figures show highly processed data after multiple steps of analyses, including GLM, support vector machine, Z-score transform, etc., the author should re-organize the part to make it clear, and probably add some equations.

To make sure there is no confusion on the steps taken in the analysis, we added a schematic overview in figure 2D. Because the individual steps are not novel by themselves, but rather the combination of steps, we believe a figure works better for clarification than equations. We also made other improvements to the figure and caption, which are now are as follows:

REBUTTAL LETTER FIGURE GOES HERE

Figure 1. Experimental and methodological framework. A. Study rationale. We studied reference frames for planning grasping movements to targets provided in two modalities: visual (top) or somaesthetic (bottom). Within each modality, reference frames were tested by aggregating evidence across four pairs of trials, either equal or distinct in gaze-centered or body-centered coordinates (right panels, black rectangles indicate pairs of trials (not shown to subject)). Two pairs, outer sides and center sides, were not used in the reference frame analysis, but used to independently define regions of interest and test for gaze direction effects. B. The two-sided evidence combination method. We specified which pairs of trials (indicated by column headers) should be distinct (?) or common (=) in gaze-centered (GC) and body-centered (BC) reference frames (1). Next, we calculated for each distinct pair the classification score (a) and for each common pair the cross-classification score (o) (2) and combined the values to obtain an evidence score per reference frame (3). C. Example classification and cross-classification procedure, within (top) and across (bottom) modalities. For each pair of trials (e.g., left w.r.t. body), a classifier was trained and cross-validated (top, first column). The resulting accuracy value represents the classification result (? or a) for the specific pair. Next, the trained classifier was tested on the four possible pairs with one of the two trial configurations replaced (e.g., gaze-center target-left replaced by gaze-right target-right, second column). In the example, if cross-classification is high (=), it gives evidence for gaze-centered coding, as the pair describing the replacement is equal in gaze-centered coordinates (third column). The procedure was also applied across modalities (bottom panel), training on one modality (first column) and testing on the other (second column) and vice versa, to test for common representations across modalities (third column). D. Schematic overview of the analysis pipeline, highlighting the most important steps in the analysis.

b) Pages 7 &amp; 8: The data was subject to a significant amount of normalization (particularly the transformation into MNI space) prior to the searchlight analysis. Some groups using multivoxel pattern analysis recommend keeping the data closer to the native functional space to reduce the number of interpolations or alternatively, if using cortical surfaces, to average within adjacent patches of the surface (but not for example across banks of a sulcus; Oosterhof et al., 2011, NeuroImage). The authors need to discuss their reasoning for using the normalization approach they did?

As we share this concern with the reviewers, we analyzed our functional data in two ways in a representative set of subjects earlier in the project. First, by normalizing the data before searchlight analysis. Second, by normalizing after searchlight analysis. The results of the two approaches was almost exactly identical. As functionally there was no difference, we decided for the current approach on the basis of practical reasons: this way, we did not have to run normalization after each searchlight run, but rather only once on the functional data beforehand, simplifying the analysis pipeline.

Also note that searchlight analysis could pick up effects across the sulcus, irrespective of when normalization is applied. To really circumvent those effects, the searchlight analysis should indeed be applied to the surface. In another, comparable, project we tested how often surface searchlight spheres pick up voxels across the sides of a sulcus. This still did happen, but again with relatively low impact on the results. From that analysis, we concluded that the functional resolution we used is too low to do the classification confidently across the surface. With higher resolution data, we agree that surface-based classification would be a good approach to take.

c) Could the authors clarify that the four main task regressors and seven additional task regressors were square-waves, spikes, and/or convolved with a hemodynamic response function. One assumes so given that it says "hemodynamic regressors" but this should be explicit and the type of hemodynamic model (e.g., two-gamma) should be specified.

We improved the specification of the hemodynamic response functions by adding two sentences:

The four hemodynamic regressors of interest were convolved with a standard hemodynamic response function (Friston et al., 2011).

Page 8, lines 24-25

And:

The seven regressors of non-interest were convolved with the same standard hemodynamic response function as the regressors of interest.

Page 9, lines 8-9

d) Page 8: Please clarify further how the first echo was used to estimate realignment parameters.

We now specify the procedure to estimate realignment parameters as follows:

The first echo was used to estimate realignment parameters, as it gives the best estimate of head motion, after which the parameters were copied to the second echo, which was used to estimate the BOLD signal (Poser et al., 2006).

Page 8, lines 4-6

e) Page 9: Could the authors please clarify the size of the voxels that formed the searchlight sphere. Initially it is confusing to the reader to state that the searchlight sphere has a "radius of two voxels" but a "sphere size" of 30 voxel. In addition, it should be stated explicitly what volume (mm^3) this corresponds to. That is, are the voxels in the native functional resolution (3.5-mm isotropic according to p. 7) or have they been resampled to another resolution during all the normalization? Better wording may be something like, "The searchlight sphere had a radius of two voxels (X mm), such that the volume of the sphere could include up to 30 voxels (X mm^3), with smaller search spheres at the cortical borders." More clarification regarding the "cortical borders" is also needed - could search volumes include voxels on opposite banks of a sulcus?

We followed the suggestion of the reviewers and now state:

All analyses were performed within local searchlight spheres (Kriegeskorte et al., 2006) with a radius of two voxels (7 mm), moved across the cortex. On average the sphere size was 30 voxels (or 1286 mm3), with smaller search spheres at the outer cortical borders.

Page 9, lines 20-22

Moreover, we updated the MRI preprocessing section:

Functional data were normalized to MNI space using the DARTEL normalization procedure (Ashburner, 2007), retaining the original dimensions of the data.

Page 8, line 10

f) Page 9: Could the authors expand on the sentence "Instead of ascribing the classification values to the center voxel of a sphere, we averaged, for each voxel, all classification results of the spheres containing the particular voxel."

We expanded the sentence in the following way:

Instead of ascribing the classification values to the center voxel of a sphere, we averaged, for each voxel, all classification results of the spheres containing the particular voxel. For example, if a voxel was included in 30 search spheres, the ascribed classification result for that particular voxel was the average of the 30 search spheres.

Page 9, lines 22-25

g) Page 11: The wording of the first two sentences of the last (incomplete) paragraph could be clearer about the specific discrimination being trained. Are you training the classifier to distinguish left-vs. right visual field when targets are on the left side of the body and then testing the same discrimination when targets are on the right side of the body?

Yes, effectively we did as the reviewers suggest. However, we did so in a step-wise manner to fully test all possible cross-classification pairs. We clarified the text (and figure, see suggestion 2) as follows:

For example, consider a classifier trained on the left w.r.t. body pair (i.e. gaze-left target-left vs. gaze-center target-left, figure 2C, top, first column). For cross-classification, the trained classifier is tested on a comparable distinction, but with one of the two trial configurations replaced: gaze-center target-left is replaced by gaze-right target-right (second column). Critically, this replacement is different in body-centered coordinates (from left to right), but is equal in gaze-centered coordinates (still left from gaze). Hence, if the cross-classification returns high performance, this can be taken as evidence for gaze-centered coding. The cross-classification evidence is ascribed to the pair describing the replacement, left w.r.t. gaze (third column). Such a replacement can be made in four ways, hence four pairs receive cross-classification evidence from one classification pair. After cross-classification was performed for all six binary classifiers, the resulting cross-classification scores were averaged per receiving pair. This average cross-classification score indicates the similarity of a pair of representations.

Pages 11-12, lines 24-8

h) Page 15: Clarify what data was used to select the local maxima in the sentence beginning "Each ROI was determined..." It didn't makes sense.

We clarified the text as follows:

Each ROI was determined on the basis of the local maxima in evidence for the independent pairs (center sides and outer sides, see figure 2A; Kriegeskorte et al., 2009), closest to the average coordinates reported in Bernier and Grafton (2010). The local maximum was determined on the basis of the group t-values for the combined classification score for the two pairs, as these two pairs are predicted to be distinct in both reference frames. The peak could be either in visual coding, somaesthetic coding, or in the sum of their scores.

Page 15, lines 5-10

4: Figure 2 is pretty complicated. Some things that could be removed to increase simplicity would be to delete the "Predictions" in 2A as it contains the same information that is presented in panel 1 of figure 2B. Panels 1 and 2 in figure 2B also contain pretty much the same information. Panel 2 could be removed and (= and o) replaced with a (y) and (? and a) replaced with an (x) Also, the labels at the bottom of figure 2C are not that intuitive. I suggest removing them and clarifying the procedure in the figure caption.

We changed figure 2 accordingly, combining it with suggestion 2a. See our response to suggestion 2a for the improved figure.

5: Apparently, a score combining classification and cross classification measures was utilized to judge whether the reference frame is closer to gaze- or body-centered. It is interesting to find that aIPS switched between them, but it is oversimplified to claim that other regions indeed are gaze- or body-centered. Throughout the results, discussion, and abstract it could be made clearer that the weighting of gaze- vs. body-centered reference frames is relative rather than absolute. I.e., it is not the case that aIPS ONLY uses an eye-centered reference frame when grasping visual targets, rather aIPS PREFERENTIALLY uses an eye-centered reference frame. The current wording (particularly in the abstract) tends to be stronger (more all-or-none) than the data suggests.

When discussing the results, we base our claims directly on the non-relative results in figure 3.5B. There, it can be seen that, according to our measures, most regions use either one or the other reference frame. SPOC is an exception, which is probably due to gaze direction tuning (see figure 7). Specifically for aIPS, there is a shift from only gaze-centered tuning for gaze-centered targets to only body-centered tuning for somaesthetic targets. We checked the text and the text is in accordance with these results.

However, as it is hard to attain both similar and distinct coding for a given pair within a region (though note magenta regions in figure 3.4), gaze-centered and body-centered tuning might have obscured each other. We now consider this option in the discussion:

In addition, dominant gaze-centered coding could obscure body-centered coding and vice-versa, as the expected effects in cortical patterns are opposite. Lastly, gaze direction effects in parietal regions might have obscured body-centered effects or induced apparent body-centered effects in the current and other studies. For example, in our study SPOC appears to also code body-centered coordinates; however the conjunction and gaze direction analysis shows this is probably an effect of gaze direction.

Pages 23, lines 18-23

To reflect this consideration and prevent an oversimplification of our results, we added the words 'predominantly' and 'mainly' at several places in the abstract and discussion.

6: The Results section (p. 16-20) could be improved by interpreting results in more coherent way rather than simply listing them individually. Moreover, many relevant parameter are missing. For example, N (trial number) should be given with SD (p. 17 top), N and statistical test should be explicitly stated with p-values (p. 19).

After careful consideration of this suggestion of the reviewers, we concluded that the current way of presenting the results leaves the most room for interpretation and own insights by the reader. We chose to leave the interpretation to the discussion.

Considering statistical parameters: indeed for the SD, the number of trials is highly relevant and we added them accordingly, as part of reporting the statistical test:

Behavorial analysis showed that participants performed the task effectively, with virtually no errors in target selection (0.05% error, range 0 - 3.05% across subjects), and matched performance across modalities, both in reaction time (reaction time to visual targets: 630 ms, SD = 189 ms; reaction time to somaesthetic targets: 626 ms, SD = 181; t(34) = -0.07, p>0.05) and movement time (movement time to visual targets: 2.465s, SD = 504 ms ; movement time to somaesthetic targets: 2.574 s, SD = 551 ms; t(34) = 0.62, p>0.05).

Page 16, lines 17-22

The specification of the test used to report p-values we now mention in the Methods section:

All group t-tests were run using a GLM with 432 scans (18 subjects * 2 modalities * 6 trial pairs * 2 tests) and 42 regressors (18 subjects + 2 modalities * 6 trial pairs * 2 tests), or a df of 432 - 41 = 391.

Page 14, lines 19-21

7: Discussion- The present finding that anterior precuneus preferentially uses a body-centered reference frame regardless of target modality is in disagreement with previous findings by Bernier and Grafton (2010) who found that this region could switch between a gaze- and body-centered reference frame depending on target modality. I want to commend the authors for acknowledging this and providing a thorough discussion of potential reasons for this difference.

We thank the reviewers for their commendation. We believe the insights gained by this apparent discrepancy gives rise to many interesting directions of further research.

8: In the Discussion, relevant studies in neurophysiology are absent. Important advances have made in recording from AIP in monkeys (e.g. by Scherberger and colleagues), providing better spatiotemporal resolutions at single-neuron level. This and other work needs to be better discussed and integrated to clarify what new findings the current work brings to our understanding of how reference frames are organised within different structures.

We now discuss the relation to the important recent work by Lehmann &amp; Scherberger (2013) on AIP:

In line with our results, neurons in the homolog area in the macaque employ predominantly gaze-centered representations when coding grasping movements to visual targets (Lehmann &amp; Scherberger, 2013). This could imply that the current results generalize from the population level to the neuronal level. However, the relatively more anterior gaze-centered zone and the relatively more posterior body-centered zone could also point to separate functional populations within the aIPS area (Romero et al., 2014). Single-neuron studies for grasping movements to somaesthetic targets, combined with repetition-suppression analyses of the current dataset, might be able to address this issue.

Page 21, lines 13-19

Minor:

There are a number of minor spelling and grammatical errors throughout. I have indicated a few below.

- Abstract, line 10: "... to explicity aggregate evidence in favor (of) and against the presence..."

- Page 6, line 2: "... until both grasping and eye movement(s) were performed..."

- Page 6, line 6: "Trials were only regarded (as) correct when the target..."

- Page 6, 3rd sentence from the end: "... 500 ms after movement o(n)set..."

- Please ensure that all acronyms are defined prior to their first use (i.e, GLM on page 7).

We thank the reviewers for the close reading and corrected all minor spelling and grammatical errors mentioned.

## References

[B1] Ashburner J. A fast diffeomorphic image registration algorithm (2007) Neuroimage 38:95–113. 10.1016/j.neuroimage.2007.07.007 17761438

[B2] Avillac M, Denève S, Olivier E, Pouget A, Duhamel JR (2005) Reference frames for representing visual and tactile locations in parietal cortex. Nat Neurosci 8:941–949. 10.1038/nn1480 15951810

[B3] Barany DA, Della-Maggiore V, Viswanathan S, Cieslak M, Grafton ST (2014) Feature interactions enable decoding of sensorimotor transformations for goal-directed movement. J Neurosci 34:6860–6873. 10.1523/JNEUROSCI.5173-13.2014 24828640 PMC4099499

[B4] Batista AP, Santhanam G, Yu BM, Ryu SI, Afshar A, Shenoy KV (2007) Reference frames for reach planning in macaque dorsal premotor cortex. J Neurophysiol 98:966–983. 10.1152/jn.00421.2006 17581846

[B5] Battaglia-Mayer A, Caminiti R, Lacquaniti R, Zago M (2003) Multiple levels of representation of reaching in the parieto-frontal network. Cereb Cortex 13:1009–1022. 12967918 10.1093/cercor/13.10.1009

[B6] Bernier PM, Grafton ST (2010) Human posterior parietal cortex flexibly determines reference frames for reaching based on sensory context. Neuron 68:776–788. 10.1016/j.neuron.2010.11.002 21092865

[B7] Beurze SM, de Lange FP, Toni I, Medendorp WP (2007) Integration of target and effector information in the human brain during reach planning. J Neurophysiol 97:188–199. 10.1152/jn.00456.2006 16928798

[B8] Beurze SM, Toni I, Pisella L, Medendorp WP (2010) Reference frames for reach planning in human parietofrontal cortex. J Neurophysiol, 104:1736–1745. 10.1152/jn.01044.2009 20660416

[B9] Binkofski F, Buxbaum LJ (2013) Two action systems in the human brain. Brain Lang 127:222–229. 10.1016/j.bandl.2012.07.007 22889467 PMC4311762

[B10] Binkofski F, Dohle C, Posse S, Stephan KM, Hefter H, Seitz RJ, Freund HJ (1998) Human anterior intraparietal area subserves prehension: a combined lesion and functional MRI activation study. Neurology, 50:1253–1259. 9595971 10.1212/wnl.50.5.1253

[B11] Björnsdotter M, Rylander K, Wessberg J (2011) A Monte Carlo method for locally multivariate brain mapping. Neuroimage 56:508–516. 10.1016/j.neuroimage.2010.07.044 20674749

[B12] Borra E, Belmalih A, Calzavara R, Gerbella M, Murata A, Rozzi S, Luppino G (2008) Cortical connections of the macaque anterior intraparietal (AIP) area. Cereb. Cortex, 18:1094–111. 10.1093/cercor/bhm146 17720686

[B13] Bremmer F, Klam F, Duhamel JR, Ben Hamed S, Graf W (2002) Visual-vestibular interactive responses in the macaque ventral intraparietal area (VIP). Eur J Neurosci 16:1569–1586. 12405971 10.1046/j.1460-9568.2002.02206.x

[B14] Brooks JL (2012) Counterbalancing for serial order carry over effects in experimental condition orders. Psychol Methods 17:600–614. 10.1037/a0029310 22799624

[B15] Buchholz VN, Jensen O, Medendorp WP (2013) Parietal oscillations code nonvisual reach targets relative to gaze and body. J Neurosci 33:3492–3499. 10.1523/JNEUROSCI.3208-12.2013 23426676 PMC6619549

[B16] Cavina-Pratesi C, Ietswaart M, Humphreys GW, Lestou V, Milner AD (2010a) Impaired grasping in a patient with optic ataxia: primary visuomotor deficit or secondary consequence of misreaching? Neuropsychologia 48:226–234. 19766131 10.1016/j.neuropsychologia.2009.09.008

[B17] Cavina-Pratesi C, Monaco S, Fattori P, Galletti C, McAdam TD, Quinlan DJ, Goodale MA, Culham JC (2010b) Functional magnetic resonance imaging reveals the neural substrates of arm transport and grip formation in reach-to-grasp actions in humans. J Neurosci 30:10306–10323. 20685975 10.1523/JNEUROSCI.2023-10.2010 PMC6634677

[B18] Chang SW, Snyder LH (2010) Idiosyncratic and systematic aspects of spatial representations in the macaque parietal cortex. Proc Natl Acad Sci U S A 107:7951–7956. 10.1073/pnas.0913209107 20375282 PMC2867917

[B19] Connolly JD, Andersen RA, Goodale MA (2003) FMRI evidence for a “parietal reach region” in the human brain. Exp Brain Res 153:140–145. 10.1007/s00221-003-1587-1 12955383

[B20] Cox D, Savoy R (2003) Functional magnetic resonance imaging (fMRI) "brain reading": detecting and classifying distributed patterns of fMRI activity in human visual cortex. Neuroimage 19:261–270. 12814577 10.1016/s1053-8119(03)00049-1

[B21] Crawford JD, Henriques DYP, Medendorp WP (2011) Three-dimensional transformations for goal-directed action. Annu Rev Neurosci 34:309–331. 10.1146/annurev-neuro-061010-113749 21456958

[B22] Culham JC, Danckert SL, DeSouza JFX, Gati JS, Menon RS, Goodale MA (2003) Visually guided grasping produces fMRI activation in dorsal but not ventral stream brain areas. Exp Brain Res 153:180–189. 10.1007/s00221-003-1591-5 12961051

[B23] Culham JC, Valyear KF (2006) Human parietal cortex in action. Curr Opin Neurobiol 16:205–212. 10.1016/j.conb.2006.03.005 16563735

[B24] Dale AM, Fischl B, Sereno MI (1999) Cortical surface-based analysis. I. Segmentation and surface reconstruction. Neuroimage 9:179–194. 10.1006/nimg.1998.0395 9931268

[B25] Davare M, Andres M, Cosnard G, Thonnard JL, Olivier E (2006) Dissociating the role of ventral and dorsal premotor cortex in precision grasping. J Neurosci 26:2260–2268. 10.1523/JNEUROSCI.3386-05.2006 16495453 PMC6674806

[B26] DeSouza JFX, Dukelow SP, Gati JS, Menon RS, Andersen RA, Vilis T (2000) Eye position signal modulates a human parietal pointing region during memory-guided movements. J Neurosci 20:5835–5840. 10908625 10.1523/JNEUROSCI.20-15-05835.2000 PMC6772534

[B27] Dinstein I, Gardner JL, Jazayeri M, Heeger DJ (2008) Executed and observed movements have different distributed representations in human aIPS. J Neurosci 28:11231–11239. 10.1523/JNEUROSCI.3585-08.2008 18971465 PMC2666623

[B94] Eickhoff SB, Stephan KE, Mohlberg H, Grefkes C, Fink GR, Amunts K, Zilles K (2005) A new SPM toolbox for combining probabilistic cytoarchitectonic maps and functional imaging data. Neuroimage 4:1325–1335. 10.1016/j.neuroimage.2004.12.034 15850749

[B28] Etzel JA, Gazzola V, Keysers C (2008) Testing simulation theory with cross-modal multivariate classification of fMRI data. PLoS One 3:e3690. 10.1371/journal.pone.0003690 18997869 PMC2577733

[B29] Etzel JA, Zacks JM, Braver TS (2013) Searchlight analysis: promise, pitfalls, and potential. Neuroimage 78:261–269. 10.1016/j.neuroimage.2013.03.041 23558106 PMC3988828

[B30] Fabbri S, Strnad L, Caramazza A, Lingnau A (2014) Overlapping representations for grip type and reach direction. Neuroimage 94:138–146. 10.1016/j.neuroimage.2014.03.017 24650596

[B31] Fattori P, Breveglieri R, Marzocchi N, Filippini D, Bosco A, Galletti C (2009) Hand orientation during reach-to-grasp movements modulates neuronal activity in the medial posterior parietal area V6A. J Neurosci 29:1928–1936. 10.1523/JNEUROSCI.4998-08.2009 19211899 PMC6666273

[B32] Fattori P, Gamberini M (2001) “ Arm-reaching” neurons in the parietal area V6A of the macaque monkey. Eur J Neurosci 13:2309–2313. 11454035 10.1046/j.0953-816x.2001.01618.x

[B33] Fattori P, Kutz DF, Breveglieri R, Marzocchi N, Galletti C (2005) Spatial tuning of reaching activity in the medial parieto-occipital cortex (area V6A) of macaque monkey. Eur J Neurosci 22:956–972. 10.1111/j.1460-9568.2005.04288.x 16115219

[B34] Filimon F (2010) Human cortical control of hand movements: parietofrontal networks for reaching, grasping, and pointing. Neuroscientist 16:388–407. 10.1177/1073858410375468 20817917

[B35] Filimon F, Nelson JD, Huang RS, Sereno MI (2009) Multiple parietal reach regions in humans: cortical representations for visual and proprioceptive feedback during on-line reaching. J Neurosci 29:2961–2971. 10.1523/JNEUROSCI.3211-08.2009 19261891 PMC3407568

[B36] Fischl B, Sereno MI, Dale AM (1999) Cortical surface-based analysis. II: inflation, flattening, and a surface-based coordinate system. Neuroimage 9:195–207. 10.1006/nimg.1998.0396 9931269

[B37] Fonov V, Evans AC, Botteron K, Almli CR, McKinstry RC, Collins DL (2011) Unbiased average age-appropriate atlases for pediatric studies. Neuroimage 54:313–327. 10.1016/j.neuroimage.2010.07.033 20656036 PMC2962759

[B38] Friston KJ, Ashburner JT, Kiebel SJ, Nichols TE, Penny WD (2011) Statistical parametric mapping: the analysis of functional brain images. New York: Academic Press.

[B39] Gallese V, Murata A, Kaseda M, Niki N, Sakata H (1994) Deficit of hand preshaping after muscimol injection in monkey parietal cortex. Neuroreport 5:1525–1529. 7948854 10.1097/00001756-199407000-00029

[B40] Galletti C, Battaglini PP, Fattori P (1995) Eye position influence on the parieto-occipital area PO (V6) of the macaque monkey. Eur J Neurosci 7:2486–2501. 8845954 10.1111/j.1460-9568.1995.tb01047.x

[B41] Galletti C, Kutz DF, Gamberini M, Breveglieri R, Fattori P (2003) Role of the medial parieto-occipital cortex in the control of reaching and grasping movements. Exp Brain Res 153:158–170. 10.1007/s00221-003-1589-z 14517595

[B42] Gamberini M, Passarelli L, Fattori P, Zucchelli M, Bakola S, Luppino G, Galletti C (2009) Cortical connections of the visuomotor parietooccipital area V6Ad of the macaque monkey. J Comp Neurol 513:622–642. 10.1002/cne.21980 19235224

[B43] Glover S (2004) Separate visual representations in the planning and control of action. Behav Brain Sci 27:3–78. 15481943 10.1017/s0140525x04000020

[B44] Grafton ST (2010) The cognitive neuroscience of prehension: recent developments. Exp Brain Res 204:475–491. 10.1007/s00221-010-2315-2 20532487 PMC2903689

[B45] Grafton ST, Fagg AH, Woods RP, Arbib MA (1996) Functional anatomy of pointing and grasping in humans. Cereb Cortex 6:226–237. 8670653 10.1093/cercor/6.2.226

[B46] Graziano MSA, Gross GC (1998) Spatial maps for the control of movement. Curr Opin Neurobiol 8:195–201. 9635202 10.1016/s0959-4388(98)80140-2

[B47] Grefkes C, Weiss PH, Zilles K, Fink GR (2002) Crossmodal processing of object features in human anterior intraparietal cortex: an fMRI study implies equivalencies between humans and monkeys. Neuron 35:173–184. 12123617 10.1016/s0896-6273(02)00741-9

[B48] Grol MJ, Majdandzić J, Stephan KE, Verhagen L, Dijkerman HC, Bekkering H, Verstraten FAJ, Toni I (2007) Parieto-frontal connectivity during visually guided grasping. J Neurosci 27:11877–11887. 10.1523/JNEUROSCI.3923-07.2007 17978028 PMC2703728

[B49] Hadjidimitrakis K, Breveglieri R, Placenti G, Bosco A, Sabatini SP, Fattori P (2011) Fix your eyes in the space you could reach: neurons in the macaque medial parietal cortex prefer gaze positions in peripersonal space. PLoS One 6:e23335. 10.1371/journal.pone.0023335 21858075 PMC3157346

[B50] Heed T, Beurze SM, Toni I, Röder B, Medendorp WP (2011) Functional rather than effector-specific organization of human posterior parietal cortex. J Neurosci 31:3066–3076. 10.1523/JNEUROSCI.4370-10.2011 21414927 PMC6623762

[B51] Hoshi E, Tanji J (2007) Distinctions between dorsal and ventral premotor areas: anatomical connectivity and functional properties. Curr Opin Neurobiol 17:234–242. 10.1016/j.conb.2007.02.003 17317152

[B52] Jeannerod M (1988) The neural and behavioural organization of goal-directed movements. Oxford, UK: Clarendon/Oxford U.P.

[B53] Jerde TA, Merriam EP, Riggall AC, Hedges JH, Curtis CE (2012) Prioritized maps of space in human frontoparietal cortex. J Neurosci 32:17382–17390. 10.1523/JNEUROSCI.3810-12.2012 23197729 PMC3544526

[B54] Kriegeskorte N, Bandettini P (2007) Combining the tools: activation- and information-based fMRI analysis. Neuroimage 38:666–668. 10.1016/j.neuroimage.2007.06.030 17976583

[B55] Kriegeskorte N, Goebel R, Bandettini P (2006) Information-based functional brain mapping. Proc Natl Acad Sci U S A 103:3863–3868. 10.1073/pnas.0600244103 16537458 PMC1383651

[B56] Kriegeskorte N, Mur M, Bandettini P (2008) Representational similarity analysis—connecting the branches of systems neuroscience. Front Syst Neurosci 2:4. 10.3389/neuro.06.004.2008 19104670 PMC2605405

[B57] Kriegeskorte N, Simmons W, Bell PSF, Baker CI (2009) Circular analysis in systems neuroscience: the dangers of double dipping. Nat Neurosci 12:535–540. 10.1038/nn.2303 19396166 PMC2841687

[B58] Lehmann SJ, Scherberger H (2013) Reach and gaze representations in macaque parietal and premotor grasp areas. J Neurosci 33:7038–7049. 10.1523/JNEUROSCI.5568-12.2013 23595761 PMC6618884

[B59] Leoné FTM, Heed T, Toni I, Medendorp WP (2014) Understanding effector selectivity in human posterior parietal cortex by combining information patterns and activation measures. J Neurosci 34:7102–7112. 10.1523/JNEUROSCI.5242-13.2014 24849346 PMC6608188

[B60] Lewis JW, Van Essen DC (2000) Corticocortical connections of visual, sensorimotor, and multimodal processing areas in the parietal lobe of the macaque monkey. J Comp Neurol 428:112–137. 11058227 10.1002/1096-9861(20001204)428:1<112::aid-cne8>3.0.co;2-9

[B61] Lingnau A, Strnad L, He C, Fabbri S, Han Z, Bi Y, Caramazza A (2012) Cross-modal plasticity preserves functional specialization in posterior parietal cortex. Cereb Cortex 24:541–549. 23118194 10.1093/cercor/bhs340

[B62] Luauté J, Schwartz S, Rossetti Y, Spiridon M, Rode G, Boisson D, Vuilleumier P (2009) Dynamic changes in brain activity during prism adaptation. J Neurosci 29:169–178. 10.1523/JNEUROSCI.3054-08.2009 19129395 PMC6664918

[B63] Macaluso E., Driver J, Frith CD (2003) Multimodal spatial representations engaged in human parietal cortex during both saccadic and manual spatial orienting. Curr Biol 13:990–999. 12814544 10.1016/s0960-9822(03)00377-4

[B64] Marzocchi N, Breveglieri R, Galletti C, Fattori P (2008) Reaching activity in parietal area V6A of macaque: eye influence on arm activity or retinocentric coding of reaching movements? Eur J Neurosci 27:775–789. 10.1111/j.1460-9568.2008.06021.x 18279330 PMC2268963

[B65] McGuire LMM, Sabes PN (2009) Sensory transformations and the use of multiple reference frames for reach planning. Nat Neurosci 12:1056–1061. 10.1038/nn.2357 19597495 PMC2749235

[B66] McGuire LMM, Sabes PN (2011) Heterogeneous representations in the superior parietal lobule are common across reaches to visual and proprioceptive targets. J Neurosci 31:6661–6673. 10.1523/JNEUROSCI.2921-10.2011 21543595 PMC3100795

[B67] Misaki M, Kim Y, Bandettini PA, Kriegeskorte N (2010) Comparison of multivariate classifiers and response normalizations for pattern-information fMRI. Neuroimage 53:103–118. 10.1016/j.neuroimage.2010.05.051 20580933 PMC2914143

[B68] Mullette-Gillman OA, Cohen YE, Groh JM (2005) Eye-centered, head-centered, and complex coding of visual and auditory targets in the intraparietal sulcus. J Neurophysiol 94:2331–2352. 10.1152/jn.00021.2005 15843485

[B69] Murata A, Gallese V, Kaseda M, Sakata H (1996) Parietal neurons related to memory-guided hand manipulation. J Neurophysiol 75:2180–2186. 8734616 10.1152/jn.1996.75.5.2180

[B70] Nakamura H, Kuroda T, Wakita M, Kusunoki M, Kato A, Mikami A, Sakata H, Itoh K (2001) From three-dimensional space vision to prehensile hand movements: the lateral intraparietal area links the area V3A and the anterior intraparietal area in macaques. J Neurosci 21:8174–8187. 11588190 10.1523/JNEUROSCI.21-20-08174.2001 PMC6763839

[B71] Pesaran B, Nelson MJ, Andersen RA (2006) Dorsal premotor neurons encode the relative position of the hand, eye, and goal during reach planning. Neuron 51:125–134. 10.1016/j.neuron.2006.05.025 16815337 PMC3066049

[B72] Pitzalis S, Sereno MI, Committeri G, Fattori P, Galati G, Tosoni A, Galletti C (2013) The human homologue of macaque area V6A. Neuroimage 82:517–530. 10.1016/j.neuroimage.2013.06.026 23770406 PMC3760586

[B73] Poser BA, Versluis MJ, Hoogduin JM, Norris DG (2006) BOLD contrast sensitivity enhancement and artifact reduction with multiecho EPI: parallel-acquired inhomogeneity-desensitized fMRI. Magn Reson Imaging 55:1227–1235. 10.1002/mrm.20900 16680688

[B74] Pouget A, Deneve S, Duhamel JR (2002) A computational perspective on the neural basis of multisensory spatial representations. Nat Neurosci Rev 3:741–747. 10.1038/nrn914 12209122

[B75] Pouget A, Snyder LH (2000) Computational approaches to sensorimotor transformations. Nat Neurosci 3:1192–1198. 10.1038/81469 11127837

[B76] Prado J, Clavagnier S, Otzenberger H, Scheiber C, Kennedy H, Perenin MT (2005) Two cortical systems for reaching in central and peripheral vision. Neuron 48:849–858. 10.1016/j.neuron.2005.10.010 16337921

[B77] Raos V, Umiltá MA, Murata A, Fogassi L, Gallese V (2006) Functional properties of grasping-related neurons in the ventral premotor area F5 of the macaque monkey. J Neurophysiol 95:709–729. 10.1152/jn.00463.2005 16251265

[B78] Rizzolatti G, Matelli M (2003) Two different streams form the dorsal visual system: anatomy and functions. Exp Brain Res 153:146–157. 10.1007/s00221-003-1588-0 14610633

[B79] Romero MC, Pani P, Janssen P (2014) Coding of shape features in the macaque anterior intraparietal area. J Neurosci 34:4006–4021. 10.1523/JNEUROSCI.4095-13.2014 24623778 PMC6705274

[B80] Rossit S, McAdam T, McLean DA, Goodale MA, and Culham, JC (2013) fMRI reveals a lower visual field preference for hand actions in human superior parieto-occipital cortex (SPOC) and precuneus. Cortex., 49:2525–2541. 10.1016/j.cortex.2012.12.014 23453790

[B81] Scott SH (2004) Optimal feedback control and the neural basis of volitional motor control. Nat Rev Neurosci 5:532–546. 10.1038/nrn1427 15208695

[B82] Selen JPJ, Medendorp WP (2011) Saccadic updating of object orientation for grasping movements. Vision Res 51:898–907. 10.1016/j.visres.2011.01.004 21232550

[B83] Sereno MI, Huang RS (2014) Multisensory maps in parietal cortex. Curr Opin Neurobiol 24:39–46. 10.1016/j.conb.2013.08.014 24492077 PMC3969294

[B95] Stelzer J, Chen Y, Turner R (2013) Statistical inference and multiple testing correction in classification-based multi-voxel pattern analysis (MVPA): random permutations and cluster size control. Neuroimage 65:69-82. 10.1016/j.neuroimage.2012.09.063 23041526

[B84] Stricanne B, Andersen RA, Mazzoni P (1996) Eye-centered, head-centered, and intermediate coding of remembered sound locations in area LIP. J Neurophysiol 76:2071-2076. 8890315 10.1152/jn.1996.76.3.2071

[B85] Todorov E (2007) Optimal control theory. In: Bayesian brain: probabilistic approaches to neural coding (Doya K, ed), pp 269–298. Cambridge, MA: MIT.

[B86] Todorov E, Jordan MI (2002) Optimal feedback control as a theory of motor coordination. Nat Neurosci 5:1226–1235. 10.1038/nn963 12404008

[B87] Toni I, Rushworth MF, Passingham RE (2001) Neural correlates of visuomotor associations. Spatial rules compared with arbitrary rules. Exp Brain Res 141:359–369. 10.1007/s002210100877 11715080

[B88] Verhagen L, Dijkerma, HC, Medendorp WP, Toni I (2012) Cortical dynamics of sensorimotor integration during grasp planning. J Neurosci 32:4508–4519. 10.1523/JNEUROSCI.5451-11.2012 22457498 PMC6622056

[B89] Verhagen L, Dijkerman HC, Grol MJ, Toni I (2008) Perceptuo-motor interactions during prehension movements. J Neurosci 28:4726–4735. 10.1523/JNEUROSCI.0057-08.2008 18448649 PMC6670443

[B90] Verhagen L, Dijkerman HC, Medendorp WP, Toni I (2013) Hierarchical organization of parietofrontal circuits during goal-directed action. J Neurosci 33:6492–6503. 10.1523/JNEUROSCI.3928-12.2013 23575847 PMC6619073

[B91] Wenderoth N, Toni I, Bedeleem S, Debaere F, Swinnen SP (2006) Information processing in human parieto-frontal circuits during goal-directed bimanual movements. Neuroimage, 31:264–278. 10.1016/j.neuroimage.2005.11.033 16466679

[B92] Zhang J, Kriegeskorte N, Carlin J, Rowe JB (2013) Choosing the rules: distinct and overlapping frontoparietal representations of task rules for perceptual decisions. J Neurosci 33:11852–11862. 10.1523/JNEUROSCI.5193-12.2013 23864675 PMC3713727

[B93] Zipser D, Andersen RA (1988) A back-propagation programmed network that simulates response properties of a subset of posterior parietal neurons. Nature 331:679–684. 10.1038/331679a0 3344044

